# Cardiac miRNA expression during the development of chronic anthracycline-induced cardiomyopathy using an experimental rabbit model

**DOI:** 10.3389/fphar.2023.1298172

**Published:** 2024-01-03

**Authors:** Michaela Adamcova, Helena Parova, Olga Lencova-Popelova, Petra Kollarova-Brazdova, Ivana Baranova, Marcela Slavickova, Tereza Stverakova, Petra Sauer Mikyskova, Yvona Mazurova, Martin Sterba

**Affiliations:** ^1^ Department of Physiology, Hradec Kralove, Czechia; ^2^ Department of Clinical Biochemistry and Diagnostics, Faculty of Medicine in Hradec Kralove and University Hospital Hradec Kralove, Hradec Kralove, Czechia; ^3^ Department of Pharmacology, Hradec Kralove, Czechia; ^4^ Department of Histology and Embryology, Charles University in Prague, Hradec Kralove, Czechia

**Keywords:** anthracyclines, chronic cardiomyopathy, cardiotoxicity, miRNA, myocardium, DNA damage response

## Abstract

**Background:** Anthracycline cardiotoxicity is a well-known complication of cancer treatment, and miRNAs have emerged as a key driver in the pathogenesis of cardiovascular diseases. This study aimed to investigate the expression of miRNAs in the myocardium in early and late stages of chronic anthracycline induced cardiotoxicity to determine whether this expression is associated with the severity of cardiac damage.

**Method:** Cardiotoxicity was induced in rabbits via daunorubicin administration (daunorubicin, 3 mg/kg/week; for five and 10 weeks), while the control group received saline solution. Myocardial miRNA expression was first screened using TaqMan Advanced miRNA microfluidic card assays, after which 32 miRNAs were selected for targeted analysis using qRT-PCR.

**Results:** The first subclinical signs of cardiotoxicity (significant increase in plasma cardiac troponin T) were observed after 5 weeks of daunorubicin treatment. At this time point, 10 miRNAs (including members of the miRNA-34 and 21 families) showed significant upregulation relative to the control group, with the most intense change observed for miRNA-1298-5p (29-fold change, *p* < 0.01). After 10 weeks of daunorubicin treatment, when a further rise in cTnT was accompanied by significant left ventricle systolic dysfunction, only miR-504-5p was significantly (*p* < 0.01) downregulated, whereas 10 miRNAs were significantly upregulated relative to the control group; at this time-point, the most intense change was observed for miR-34a-5p (76-fold change). Strong correlations were found between the expression of multiple miRNAs (including miR-34 and mir-21 family and miR-1298-5p) and quantitative indices of toxic damage in both the early and late phases of cardiotoxicity development. Furthermore, plasma levels of miR-34a-5p were strongly correlated with the myocardial expression of this miRNA.

**Conclusion:** To the best of our knowledge, this is the first study that describes alterations in miRNA expression in the myocardium during the transition from subclinical, ANT-induced cardiotoxicity to an overt cardiotoxic phenotype; we also revealed how these changes in miRNA expression are strongly correlated with quantitative markers of cardiotoxicity.

## Introduction

Anthracycline (ANT) antibiotics (e.g., doxorubicin or daunorubicin) are essential to various anticancer therapies for many haematological and solid malignancies (e.g., acute leukemias, lymphomas, breast cancer and sarcomas). However, all of the clinically used ANTs are also known to involve a risk of chronic cardiotoxicity, which may result in dilated cardiomyopathy and heart failure (HF) months or years after the chemotherapy. The growing number of long-term cancer survivors who received ANTs necessitates a science-led discussion on the extent of cardiotoxicity caused by ANTs and how the negative impacts of cancer treatment can be minimised. Unfortunately, the mechanisms underlying the development of ANT-induced cardiotoxicity remain incompletely understood, which hampers effective targeted measures for the prevention of adverse side effects. Topoisomerase IIB has recently been proposed to be a key molecular target for ANTs in the heart (Zhang et al., 2012). The detrimental interaction between ANTs and topoisomerase IIB triggers DNA damage, yet the subsequent biochemical events that lead to cardiotoxicity and potential HF remain unclear (Ewer and Ewer, 2015; Sawicki et al., 2021). However, it is important to note that many alternative mechanisms for ANT-induced cardiotoxicity have been proposed (Renu et al., 2018; Sawicki et al., 2021).

MicroRNAs (miRNAs) play a unique role in the regulation of gene expression and can impart tremendous impacts on the normal and pathological physiology of most organ systems (O'Brien et al., 2018; Ha, 2011; Ilieva M et al., 2022). A thorough analysis of the role of miRNAs in ANT-induced cardiotoxicity could have significant clinical relevance yet has not yet been realized. MiRNAs are small (approximately 22 nucleotides), non-coding, endogenous, single-stranded-RNA molecules that regulating gene expression by inhibiting translation or promoting mRNA degradation (Bartel, 2004). miRNAs are expressed in all mammalian cells and appear to be highly conserved across various species. It has been predicted that a single miRNA can have more than 1,000 target genes, and that multiple miRNAs can regulate a single protein-coding gene. Thus, miRNAs should be viewed as regulators of cellular function, or a certain cellular program, rather than regulators of a single gene (Wang, 2010). MiRNAs have a vital role in the physiological function of the heart as they influence both the electrical and mechanical properties of the myocardium. Correspondingly, distinct alterations in the miRNA profile have been associated with cardiac remodelling, fibrosis and heart failure (Thum et al., 2007; Harada et al., 2014; Chistiakov et al., 2016; Chen et al., 2017; Dhingra and Vasan, 2017; Peters et al., 2020; Gargiulo et al., 2023).

In the last few years, several experimental *in vitro* studies employing rat neonatal cardiomyocytes, human-induced pluripotent stem cell-derived cardiomyocytes (iPSC), or cardiomyocyte-related cell lines were performed to describe the changes in miRNAs expression associated with ANT-induced cardiotoxic effects (Chaudhari et al., 2016; Holmgren et al., 2016; Ruggeri et al., 2018a; Chen et al., 2021). However, the results obtained are relatively heterogeneous which may reflect employment of different concentrations of ANT, schedule of cell exposure as well as cellular model. Moreover, *in vitro* data cannot reliably describe the complex changes which develop slowly in clinical settings over many weeks or months. Indeed, several rodent *in vivo* studies as well as clinical trials have been performed in the past few years (see the review of Piegari et al., 2016; Ruggeri et al., 2018a; Pereira et al., 2020; Chen and Xu, 2021; Brown et al., 2022). However, these studies have typically only concentrated on a few “cardiac enriched” miRNAs (e.g., miR-208, miR-1, and miR-133), while the presented results have been highly variable, possibly due to differences in dosage schedules, ANT administration, and the duration of the treatment (Nishimura et al., 2015; Oliveira-Carvalho et al., 2015; Rigaud et al., 2017; Alves et al., 2022). A systematic review of the miRNAs that are currently used as plasma biomarkers for chemotherapy-induced cardiotoxicity in breast cancer patients suggest that three miRNA markers (miR-29a, miR-34a and miR-423) can be used as general cardiotoxicity indicators but should be supplemented with data concerning miR-1, miR-499 and miR-122 for patients receiving doxorubicin (Brown et al., 2022).

It is important to state that miRNA expression is typically transient rather than constant. As such, miRNA expression can dynamically change to reflect the cellular and molecular alterations taking place in the myocardium. Hence, it can be assumed that miRNA expression will vary with respect to the stage of cardiotoxicity development; in the later stages, the expression profile can be impacted by the severity of the cardiotoxic insult and resulting myocardial remodelling. Results from cardiological examinations in the clinical setting that demonstrate the correlation between miRNA changes and the cardiac status would provide definitive proof for the theory that miRNAs are important in the development of cardiotoxicity. However, such an analysis is unfeasible, as evidence of miRNA levels would be limited to plasma samples, in which the cardiac miRNA signature would be mixed with the miRNA response to the tumour and the responses in other organs. Furthermore, the mechanistic insights of studies performed in the clinical environment would likely be hampered by variation in the therapeutic regiments of different patient populations (Ruggeri et al., 2018b). Carefully designed *in vivo* studies using chronic animal models of ANT cardiotoxicity, which are characterised by a slow development of cardiotoxicity over repeated cycles of ANT chemotherapy, could overcome these limitations. Unfortunately, there is limited data from well-established translational models; moreover, the relationship between the myocardial miRNA expression profile and stage of cardiotoxicity has not yet been properly studied. An analysis of the global miRNA changes associated with full-blown ANT cardiotoxicity may provide interesting insights, but the results would be inherently impacted by general pathophysiological changes occurring in the heart independent of original aetiology. Hence, the ability to compare miRNA expression differences between an early phase of cardiotoxicity development—during which damage is only detectable through sensitive biomarkers—and later stages of cardiotoxicity that involve significant changes to cardiac function and morphological remodelling of the myocardium, is of particular importance.

In the present study, we aimed to describe changes of the miRNA expression profile in the left ventricular (LV) myocardium during two distinct stages of ANT-induced cardiomyopathy (early subclinical cardiotoxicity vs. fully developed cardiotoxicity); the correlations between these findings and quantitative parameters of toxicity (biomarkers of cardiac damage and cardiac function examination) were then assessed using a well-validated rabbit model.

## Materials and methods

### Study design and animal treatment

All of the animal handling methods and related procedures were approved and supervised by the Animal Welfare Body of the Faculty of Medicine in Hradec Kralove, Charles University. The research conformed to the Guide for the Care and Use of Laboratory Animals (National Academy Press, 1996).

The animal model of chronic DAU-induced cardiomyopathy in rabbits has been extensively characterized and described in the literature (Adamcová et al., 1999; Šimůnek et al., 2003). Furthermore, plasma concentrations of DAU in this model are in the clinically relevant range (Kollárová-Brázdová, et al., 2021). Based on previous experience and related published data, two intervals were purposely selected for the analysis of microRNA during the development of chronic cardiotoxicity in the present study.

The first analysis was done after five weekly DAU cycles (cumulative dose ∼ 250 mg/m^2^) when the first signs of the chronic cardiotoxicity, i.e., significantly increased levels of plasma cTnT, have been previously found, but without any significant concomitant change in systolic function as determined by non-invasive examinations (Sterba et al., 2007; Jirkovský et al., 2013; Adamcova et al., 2015). The second analysis was performed after ten weekly DAU cycles (cumulative dose ∼ 500 mg/m^2^), which is known from previous studies to induce significant LV systolic dysfunction and all the typical histopathological hallmarks of chronic ANT cardiotoxicity (Šimůnek et al., 2003).

The study was carried out on four groups of young adult New Zealand male rabbits (initial weight 3.15 ± 0.12 kg). The first group (n = 8) received intravenous daunorubicin (DAU, 3 mg/kg, as a hydrochloride salt, pharmaceutical grade, Euroasia’s Group Inc., Mumbai, India), once a week for 5 weeks, while the second group (control; n = 7) received saline solution (1 mL/kg, on the same schedule). The third (n = 8) and fourth (n = 7) groups received the same weekly treatments of DAU and saline solution as described above, respectively, but the treatment lasted 10 weeks instead of 5 weeks.

Non-invasive procedures were performed under light anaesthesia, which was induced by the intramuscular administration of a mixture of ketamine (30 mg/kg) and midazolam (2.5 mg/kg). A pentobarbital solution [4% (w/w), i.v.] was administered prior to invasive hemodynamic measurements, which were performed at the end of the experiment, and also used for animal overdose. During necropsy, the heart was rapidly excised, briefly washed, and then retrogradely perfused with ice-cold saline using a syringe. Transversal sections of the whole heart were cut for histological analysis, while the rest of the left ventricular (LV) was frozen in liquid nitrogen, pulverised under liquid nitrogen, and stored at −80°C.

### Examination of cardiac function

LV systolic function was examined by both echocardiography and LV catheterisation. Echocardiography was performed weekly in the 8th-10th weeks of the experiments, as well as at the scheduled end of the study, using a Vivid 4 ultrasound device equipped with a 10 MHz probe (GE Healthcare, Chalfont St. Giles, United Kingdom). The left parasternal approach was employed to perform guided M-mode examinations in the long and short axis views, and LV dimensions (end-systolic and end-diastolic) were also determined. At least three independent measurements, with four heart cycles evaluated in each, were used to determine fractional shortening (LV FS) as an index of systolic function. At the end of the study, invasive LV hemodynamic measurements were performed *via* carotis sinistra using a Micro-Tip pressure catheter (2.3F, Millar Instruments, Houston, TX, United States) connected to a data acquisition system (Powerlab, ADInstruments, Dunedin, New Zealand). The first derivative of the LV pressure change measured during isovolumic contraction (index of systolic function dP/dt_max_) was calculated using Chart 5.4.2 software (ADInstruments).

### Determination of cardiac troponins in plasma

Blood samples (∼1.0 mL) were obtained from a marginal ear vein before the 1st and 5th or 10th drug administration, respectively. Blood was collected into a BD Vacutainer (BD Biosciences, Franklin Lakes, NJ) containing lithium heparin; plasma samples were stored at −80°C until further analyses.

Concentrations of cTnT were determined using an Elecsys Tropo T hs STAT (Roche Diagnostics, Basel, Switzerland) with a detection limit of 0.003 μg/L.

### Histopathological evaluation

The transverse paraffin sections of heart ventricles (7 µm thick) were routinely processed and stained with H&E and Masson’s blue trichrome (MT) for standard histological evaluation.

In the case of Masson’s blue trichrome, slides were stained with Ponceau 2R/Acid Fuchsin solution and Aniline Blue solution. Masson’s blue trichrome is mainly used for detecting early changes in the tinction of myocardial cells (increased eosinophilia of their cytoplasm) and for selective staining of collagen fibers (dark blue).

### Quantitative real-time PCR of miRNAs

Total RNA, including miRNAs, was isolated from approximately 50 mg of homogenised LV tissue using a miRNeasy Mini Kit (Qiagen, Hilden, Germany) according to the manufacturer’s protocol. The frozen samples, which had been kept at −80°C prior to analysis, underwent mechanical tissue homogenisation using a MagNA Lyser Instrument (Roche, Basel, Switzerland). The RNA was eluted in 50 µL of DNase/RNase-Free Water. For plasma samples, total RNA was isolated from 200 µL of rabbit plasma using a miRNeasy Mini Kit (Qiagen) with the addition of glycogen in the lysis step as the small RNA carrier/co-precipitant. The RNA was eluted in 14 µL of DNase/RNase-Free Water. RNA concentration and purity were determined spectrophotometrically on a NanoDrop ND 1000 (Thermo Fisher Scientific, Waltham, MA) or a DS-11 FX Spectrophotometer/Fluorometer (DeNovix Inc., Wilmington, DE) by measuring optical density at 260 nm and 280 nm, along with the A260/280 ratio. After isolation, the samples were immediately processed or stored at −80°C.

cDNA synthesis was performed using a TaqMan^®^ Advanced miRNA cDNA Synthesis Kit with universal reverse transcription primers (Applied Biosystems, Foster City, CA) according to the manufacturer’s protocol; the reaction required 8–10 ng of total RNA.

The differential expression of miRNAs in a limited number of samples (n = 4 for each group) was screened using TaqMan^®^ Advanced miRNA Array Cards (Applied Biosystems), which were 384-well microfluidic cards prepared with dried-down TaqMan^®^ Advanced miRNA Assays. TaqMan^®^ Advanced miRNA Human A and B Cards were selected for the analyses and enabled the quantification of 754 unique miRNAs. To determine the most suitable endogenous control, we used a TaqMan^®^ Advanced miRNA Human Endogenous Controls Card (Applied Biosystems) with two pools of samples (treated vs. controls). Data analysis was performed using Thermo Fisher Connect Platform (https://apps.thermofisher.com/apps/spa/#/dashboard); more specifically, the Relative Quantification app using Global Mean for data normalization and manually corrected for low amplification or wrong automatic threshold detection.

Based on the results from the TaqMan^®^ Advanced miRNA Human A and B Cards, along with information from the literature, 32 miRNAs were selected for targeted analysis using quantitative real-time PCR with specific assays (TaqMan^®^ Advanced miRNA Assays, Applied Biosystems)—Table 1. In the assay, two miRNAs (hsa-miR-361-5p and hsa-miR-30c-5p) were used as endogenous controls, and the results were analysed in RefFinder, a web-based comprehensive tool developed for evaluating and screening reference genes from extensive experimental datasets. This tool integrates all of the currently available major computational programs, i.e., geNorm, Normfinder, BestKeeper, and the comparative Delta-Ct method.

**TABLE 1:** Selected miRNAs for targeted qPCR analysis.

Selected miRNAs for targeted qPCR analysis			
let-7e-5p	miR-21-3p	miR-34c-5p	miR-193a-3p
let-7f-2-3p	miR-21-5p	miR-99a-5p	miR-223-3p
let-7f-5p	miR-27b-3p	miR-133a-3p	miR-433-3p
miR-9-3p	miR-28-5p	miR-142a-3p	miR-497-5p
miR-16-5p	miR-29b-3p	miR-144-3p	miR-499a-3p
miR-19b-3p	miR-34a-3p	miR-146-5p	miR-504-5p
miR-20a-5p	miR-34a-5p	miR-152-3p	miR-1249-3p
miR-20b-5p	miR-34b-3p	miR-155-5p	miR-1298-5p

All of the real-time PCR reactions were performed in triplicates; the reaction volume was 10 μL, which included 2.5 µL of the cDNA sample. The reaction conditions were set according to the manufacturer’s protocol and involved enzyme activation at 95°C for 20 s, followed by 40 cycles of denaturation at 95°C for 3 s, and annealing/extension at 60°C for 30 s. The obtained data were analysed in the Rotor Gene Q Series Software (Qiagen). The relative expression of each miRNA was determined using the 2^−ΔΔCT^ method, with expression levels of miR 361-5p used for data normalization.

### Western blotting of p53

The Western blot analysis of p53 in LV myocardial samples was performed as described previously (Kollárová-Brázdová et al., 2021). Briefly, proteins in the LV myocardial samples were separated by SDS-PAGE using TGX Stain-Free precast gels (Bio-Rad, Hercules, CA). Immunodetection was performed with a mouse anti-p53 purified primary antibody (BP53-12; Exbio Praha a.s., Prague Czech Republic; dilution 1:1000) and an anti-mouse secondary antibody (P0447, Polyclonal Goat Anti-Mouse Immunoglobulin/HRP; DAKO Denmark A/S, Glostrup, Denmark; dilution 1:1000). A BM Chemiluminescence Western blotting Substrate (Roche) and Fusion Solo S imager coupled with a CCD camera (Vilber Lourmat GmbH, Eberhardzell, Germany) were used for signal detection. The results were normalized based on the total protein levels observed on the Stain-Free precast gels (Bio-Rad).

### Statistical and bioinformatics analyses

SigmaStat 3.5 software (SPSS Inc., Chicago, IL) was used for statistical analyses. The statistical significance of between-sample differences was assessed using either a one-way ANOVA or Kruskal–Wallis ANOVA test based on data characteristics. Data related to the functional parameters of LV are presented as the mean value ± SD, while results from cTnT measurements are presented as the median value with boxes and whiskers representing the interquartile range and 5th–95th percentiles. MiRNA data are reported as the fold change in comparison to the corresponding control (mean ± SD) and the statistical significance of differences was evaluated using a *t*-test. Bioinformatic analyses were performed using miRTargetLink v.2.0. (Kern et al., 2021). and FunRich Tool v.2.1.2. Correlation analyses between individual miRNAs and cTnT, FS and dP/dt_max_ were performed using either Spearman’s or the Pearson correlation coefficient according to data characteristics. GraphPad Prism 8.00 (GraphPad Software, Boston, MA) was used to prepare the graphical representations of the results.

## Results

Repeated DAU treatment did not induce any premature death up until the fifth week of the study, but animal mortality (25%) did occur in the second half of the 10-week experiment. The necropsy examinations performed in these animals found dilation of both cardiac ventricles and hydrothorax/ascites as signs of blood congestion.

### Plasma cTnT concentration

**FIGURE 1:**
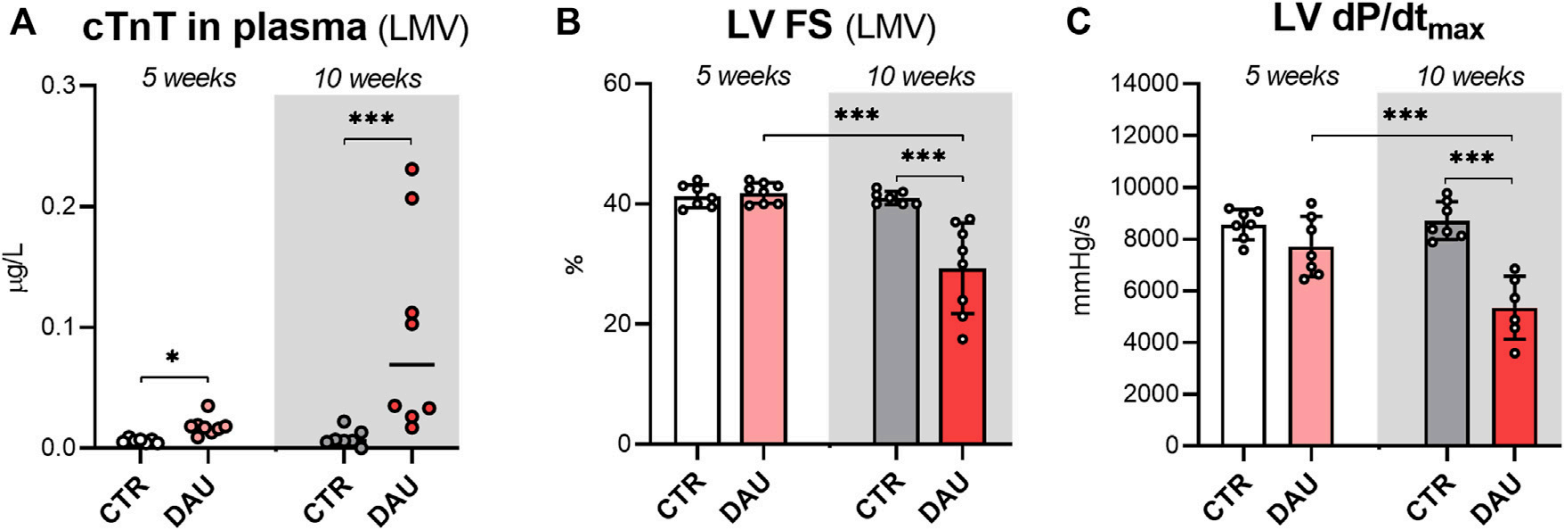
Levels of cardiac troponin T (cTnT), a biomarker of cardiac damage, and examination of systolic function in rabbits after 5 and 10 weekly doses of DAU. **(A)** Plasma concentrations of cardiac troponin T as a marker of cardiac damage. **(B)** Echocardiography-assessed left ventricular systolic function (FS - fractional shortening). **(C)** Catheterization-assessed index of the left ventricular systolic function (dP/dtmax). The results are shown as median values, with boxes and whiskers representing the interquartile range and the 5th–95th percentile, respectively **(A)**, or as the mean value ± SD **(B,C)**. Significant differences are displayed as follows: *p* < 0.05 (*); *p* < 0.01 (**); *p* < 0.001 (***). 5 weeks corresponds to 5x DAU 3 mg/kg/week (cumulative dose ∼ 250 mg/m^2^); 10 weeks corresponds to 10x DAU 3 mg/kg/week (cumulative dose ∼ 500 mg/m^2^).

The animals treated with DAU showed moderately higher plasma concentrations of cTnT after 5 weeks of DAU administration relative to the control animals (median values: 0.018 vs. 0.006 μg/L; *p* < 0.001). In the 10-week experiment, the animals receiving DAU showed significantly higher plasma concentrations of cTnT at the end of the experiment relative to control animals (median values: 0.069 vs. 0.006 μg/L; *p* < 0.05)—Figure 1A.

### Examination of LV function

The echocardiographic examination of LV fractional shortening (LV FS) did not reveal any significant differences between the DAU and control groups after 5 weeks of treatment (41.8% ± 1.7% vs. 41.3% ± 1.9%, respectively) (Figure 1B). Similarly, no significant between-group differences were observed for the index of LV contractility (dP/dt_max_) obtained by invasive examination (7710 ± 1158 mmHg/s vs. 8560 ± 582 mmHg/s, respectively, *n.s.*) (Figure 1C).

After 10 weeks of DAU administration, both non-invasive and invasive examinations confirmed a significant impairment of LV systolic function. LV FS was significantly reduced in the DAU group compared to the control group (29.3% ± 8.2% vs. 41.0% ± 1.0%, respectively; *p* < 0.001) (Figure 1B). Similarly, the DAU-treated animals showed an index of contractility dP/dt_max_ that was 38% higher than what was observed for the control group (vs. 8714 ± 727 vs. 5341 ± 1222 mmHg/s, respectively; *p* < 0.001)—(Figure 1C).

### Histological evaluation of the LV myocardium

**FIGURE 2:**
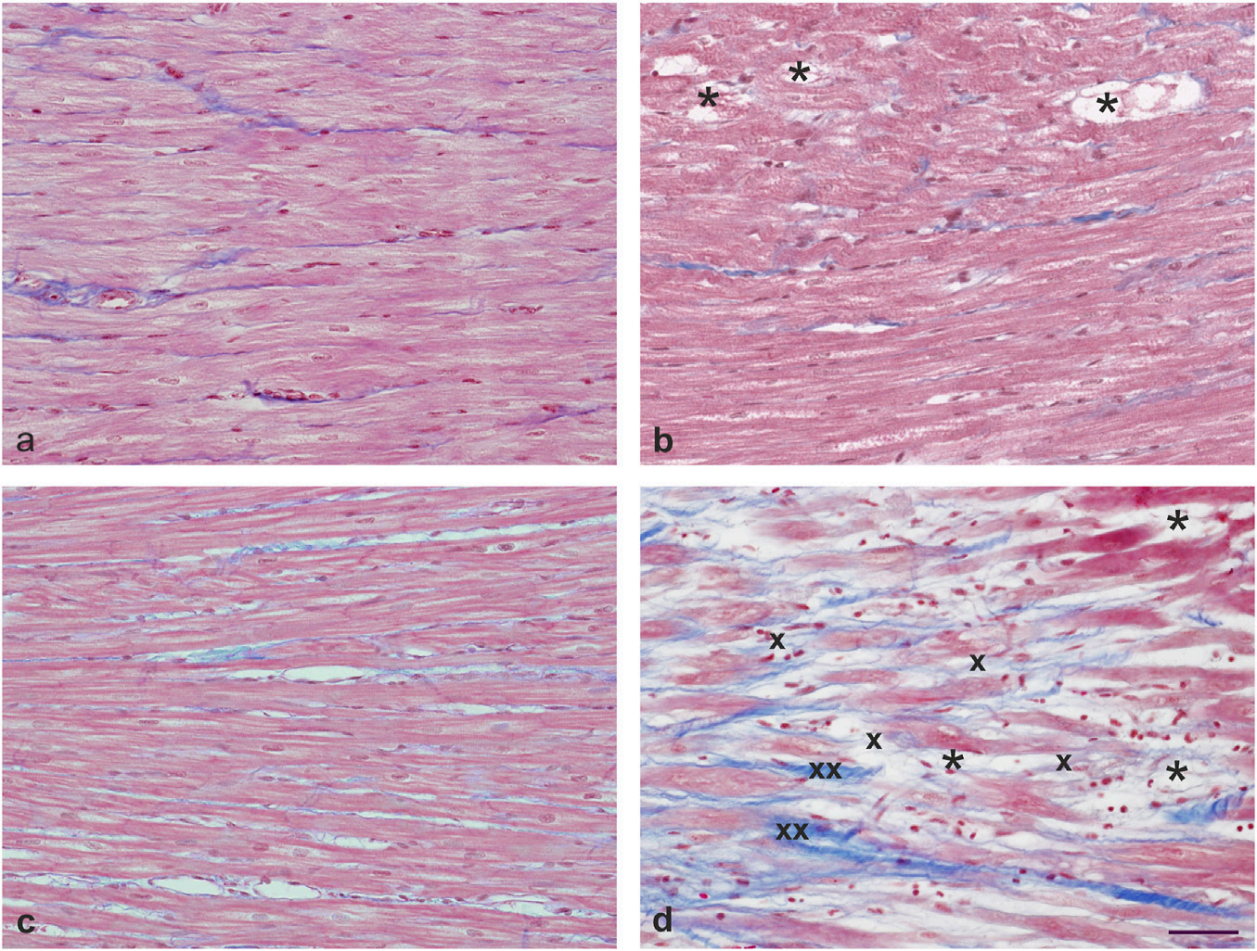
Histological analysis of LV myocardium. Comparison of the structure of the LV myocardium after five **(B)** and 10 weeks of daunorubicin (DAU) administration, **(D)** confirmation of two markedly different stages of chronic anthracycline-induced cardiomyopathy development, while normal structure of the myocardium was found in both age-matched controls **(A,C)**. After 5 weeks of ANT administration **(B)**, only initial stages of developing cardiomyopathy were seen, namely, a single cardiomyocyte with vacuolized cytoplasm and loss of myofibrils (*) could be found inside otherwise mostly intact myocardium. In contrast, 10 weeks ANT treatment **(D)** resulted in the development of distinct focal damage of the myocardium, manifested mainly by advanced stages of cell degeneration (necrosis)—i.e., gradually increasing loss of myofibrils and vacuolization of the cytoplasm (*), resulting in cell death. Necrotic cardiomyocytes were found to be gradually replaced by fibrotic scar tissue, more specifically, blue-stained collagen fibres (collagen type I) that form a fine mesh at first (x), but gradually progress to thick bundles (xx). Masson’s blue trichrome staining. Bar 50 μm.

The comparison of LV myocardial structure five (Figure 2B) and 10 weeks after the administration of DAU (Figure 2D) confirmed two distinct stages in the development of chronic ANT cardiomyopathy, while both age-matched controls demonstrated normal myocardial structure (Figures 2A, C). Only the initial stages associated with the development of cardiomyopathy were observed after 5 weeks of DAU treatment (Figure 2B). More specifically, only a few cardiomyocytes with a vacuolized cytoplasm and loss of myofibrils (*) could be found inside an otherwise mostly intact myocardium. No distinct fibrosis, infiltration or oedema could be recognised. In contrast, 10 weeks of DAU treatment (Figure 2D) resulted in the development of distinct focal damage to the myocardium, which mainly manifested as advanced stages of cell degeneration (necrosis), i.e., gradually increasing loss of myofibrils and vacuolization of the cytoplasm (*), resulting in cell death. The necrotic cardiomyocytes were gradually replaced by fibrotic scar tissue, e.g., blue-stained collagen fibres (collagen type I), which initially formed a fine mesh (x), but gradually evolved into thick bundles (xx).

### Analysis of miRNA expression in the LV myocardium

The miRNA screening, which employed TaqMan^®^ Advanced miRNA Array Cards, identified significant changes in the miR-34 family in both animal groups treated with DAU for 5 weeks and 10 weeks. Notably, miR-34a-5p was found to be upregulated in the myocardium, with a fold-change relative to the control group of 4.49 (corrected *p* = 0.001) and 33.16 (corrected *p* = 0.016) after five and 10 weeks of DAU treatment, respectively. After the 10-week treatment period, mir-34c-5p was also upregulated (fold change 14.85, corrected *p* = 0.032). In addition, miR-1298-5p was significantly upregulated after 10 weeks of DAU treatment (fold change 47.40, corrected *p* = 0.014).

**FIGURE 3:**
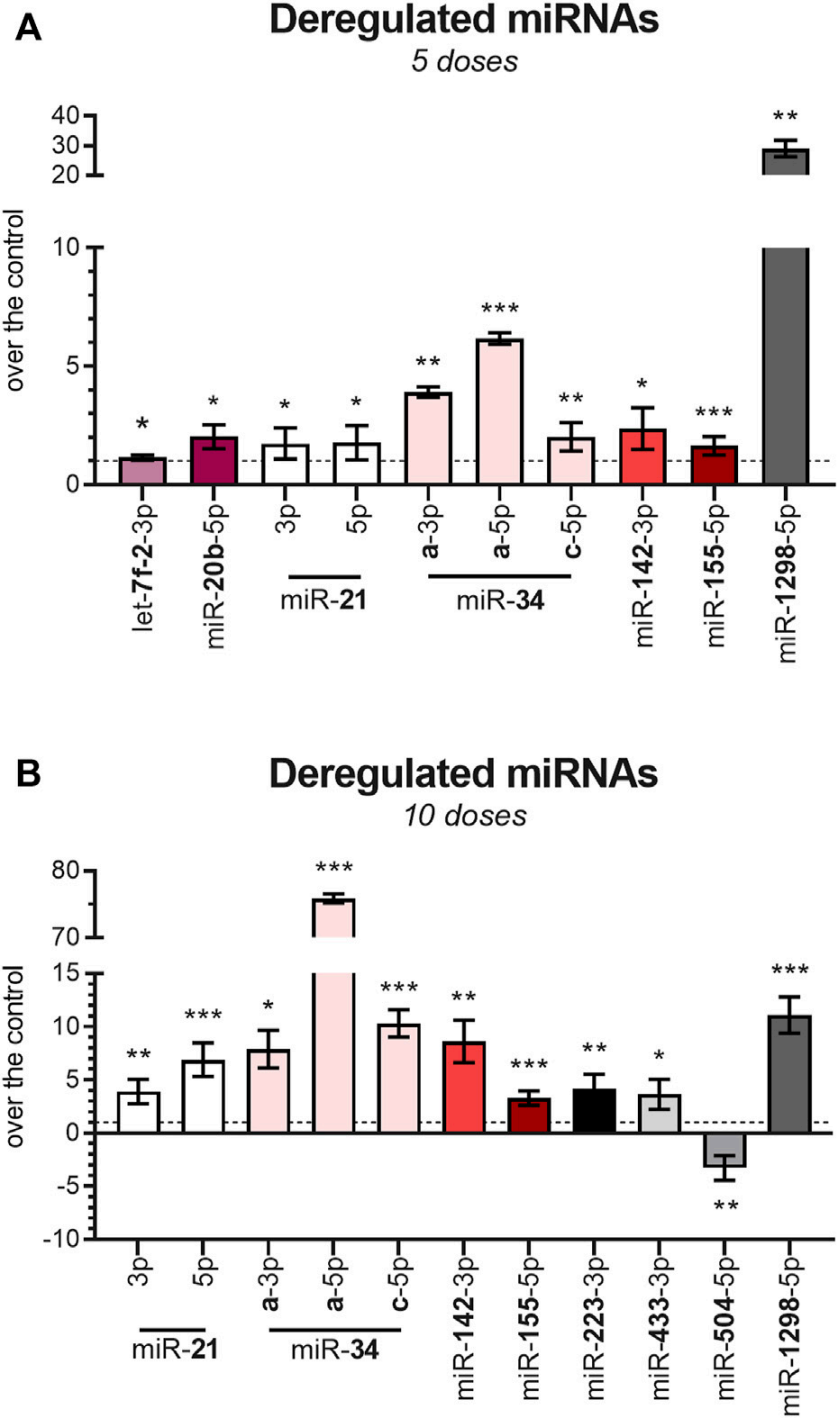
Deregulation of miRNA expression after DAU treatment **(A)** miRNAs which showed deregulation after 5 weeks of DAU treatment. **(B)** miRNAs which showed deregulation after 10 weeks of DAU treatment. Results are given as the fold change relative to the control group. Data are presented as the mean value ± SD. The statistical significance of changes as compared with the control group is displayed as follows: *p* < 0.05 (*); *p* < 0.01 (**); *p* < 0.001 (***).

**FIGURE 4:**
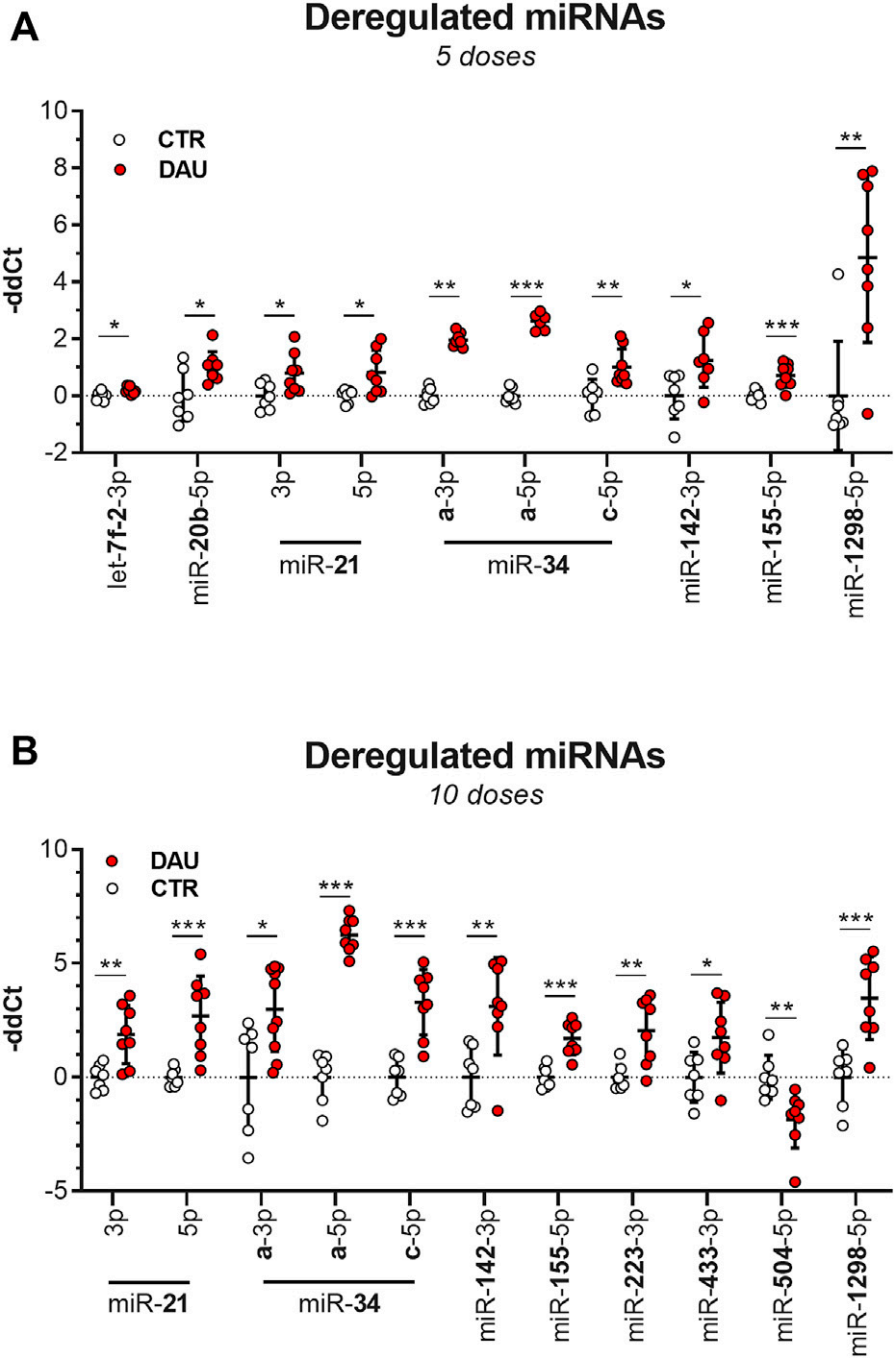
Deregulation of miRNA expression after DAU treatment (the scatter plot showing all the individual values) **(A)** miRNAs which showed deregulation after 5 weeks of DAU treatment. **(B)** miRNAs which showed deregulation after 10 weeks of DAU treatment. Results are given as the fold change relative to the control group. Data are presented as the mean value ± SD. The statistical significance of changes as compared with the control group is displayed as follows: *p* < 0.05 (*); *p* < 0.01 (**); *p* < 0.001 (***).

Based on these findings, along with what has been reported in previous literature, a panel of 32 miRNAs was selected for the full-fledged quantitative analysis of expression across all samples (RT-qPCR). After 5 weeks of DAU treatment, 10 miRNAs were significantly upregulated relative to the control group, with miR-1298-5p showing the strongest upregulation (29-fold change, *p* < 0.01) (Figure 3A; Figure 4A reporting individual values). It is noteworthy that three members of miR-34 family also showed significant upregulation relative to the control group, with miR-34a-5p showing the second most prominent change (fold change 6.2, *p* < 0.001). miR-let-7f-2-3p (*p* < 0.05) and miR-20b-5p (*p* < 0.05) were also found to be dysregulated at earlier stages of DAU treatment, but this dysregulation did not appear in later phases of cardiomyopathy development.

Following 10 weeks of DAU treatment, only miR-504-5p (*p* < 0.01) was significantly downregulated, and 10 miRNAs demonstrated significant upregulation, with miR-34a-5p (76-fold change) showing the most significant change (Figure 3B; Figure 4B reporting individual values). The two other studied members of the miR-34a family (miR-34a-3p and miR-34c-5p) were also significantly upregulated in the DAU group relative to the control group, but to a lower extent (7.9-fold and 10.3-fold, respectively).

Several miRNAs showed statistically significant differential expression with respect to the length of DAU treatment (5 vs. 10 weeks). The members of the miR-34 family showed higher levels of expression as the experiment progressed; this was particularly evident for miR-34a-5p and miR-21, and—to a lesser extent—miR-142-3p and miR-155-5p. An opposite trend was observed for miR-1298-5p, which showed a 3-fold decrease from the five-week to 10-week experiment (*p* < 0.001). Expression of the other studied miRNAs also differed between the two study time-points. At the 10-week time-point, miR-223 (*p* < 0.01), miR-433-3p (*p* < 0.05), and miR-504-3p (*p* < 0.01) had become dysregulated in comparison to the five-week time-point, while the significant expression changes seen for miR-let-7f-2-3p and miR-20b-5p at the earlier time-point had disappeared at the 10-week time-point. For miR-34a and miR-21, co-expression of mature miRNAs from the 5′ and 3′ arms of pre-miRNA precursors were observed. In most cases, only one species remains while the complementary species is degraded, but the coexistence of miRNA-5p and miRNA-3p species has been previously reported (Choo et al., 2014).

### Correlations between the levels of various miRNAs and cardiotoxicity parameters

**FIGURE 5:**
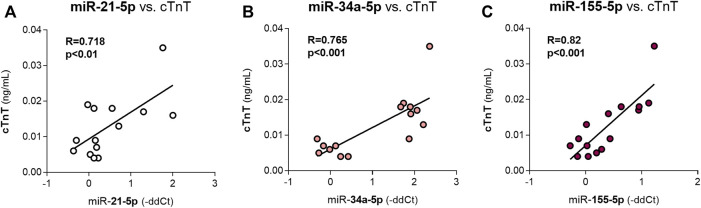
Correlations between the expression levels of miR-21-5p, miR-34a-5p, miR-155-5p and cTnT levels (a marker of cardiac damage) after 5 weeks of DAU treatment R: the coefficient of correlation. The statistical significance of changes as compared to the control group is displayed as follows: *p* < 0.05 (*); *p* < 0.01 (**); *p* < 0.001 (***).

After 5 weeks of DAU administration, the myocardial expression levels of most miRNAs were significantly positively correlated with the plasma concentrations of cTnT (Table 2), which is a sensitive marker of cardiac microinjury. The miR-21-5p, miR- 34a-5p and miR-155 were strongly correlated with this parameter (Figures 5A–C). These miRNAs are mainly related to DDR. No significant correlations were found between cTnT levels and miR-let7f-2-3p, miR-21-3p or miR-34c-5p expression. Correlations with parameters of cardiac function were not analysed because these parameters did not show any significant change due to the DAU treatment in this part of the study.

**TABLE 2:** Correlation between selected miRNAs and plasma level of cTnT as a marker of cardiac damage after the 5th week of DAU treatment.

	cTnT		
	R	*p*	
miR-**20b**	0.599	0.05	*
miR-**21**-3p	0.696	0.01	**
miR-**21**-5p	0.718	0.01	**
miR-**34a**-3p	0.765	0.01	**
miR-**34a**-5p	0.765	0.001	***
miR-**142**	0.570	0.05	*
miR-**155**	0.820	0.001	***
miR-**1298**	0.630	0.05	*

**FIGURE 6:**
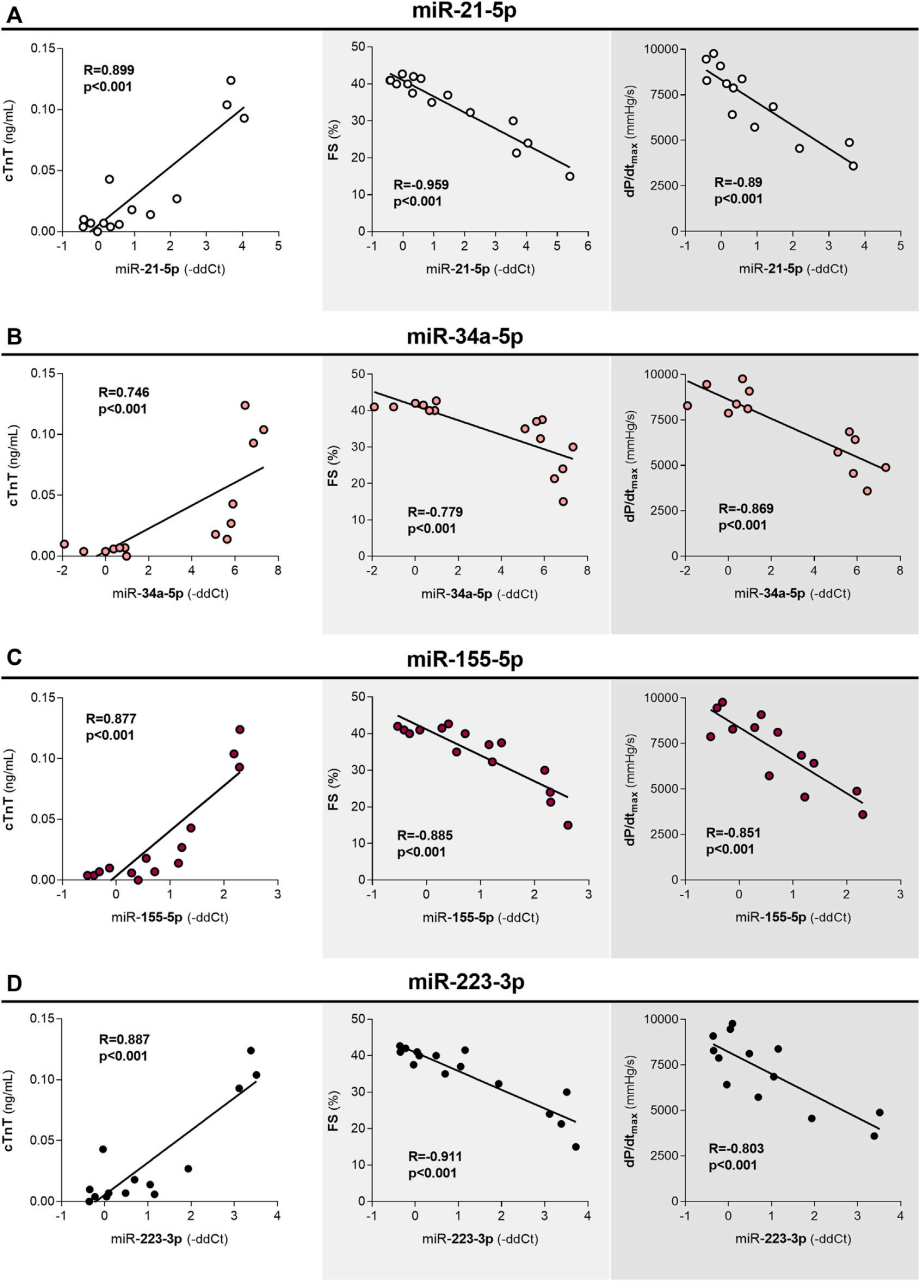
Correlations between the expression of miRNAs and cTnT levels, and between the expression of miRNAs and functional parameters of LV after 10 weeks of DAU treatment cTnT - plasma concentrations of cardiac troponin T, a marker of cardiac damage. FS - fractional shortening - echocardiography-assessed left ventricular systolic function. dP/dt_max_ - catheterization-assessed index of left ventricular systolic function. R: the coefficient of correlation. The statistical significance of changes as compared to the controls is displayed as follows: *p* < 0.05 (*); *p* < 0.01 (**); *p* < 0.001 (***).

After 10 weeks of DAU treatment, except for miR-433-3p, all of the miRNAs that demonstrated differential expression were significantly correlated with cTnT levels (the correlations were positive in all other cases except miR-504-3p). Moreover, miR-34a-3p expression was only weakly correlated with cTnT levels. The expression levels of most of these miRNAs were also correlated with parameters of systolic function (LVFS and dP/dt_max_); an exception was miR-34a-3p, as no significant correlation was found, while miR-433 expression was only correlated with FS (Table 3). The strongest observed correlations were between miR-21-5p, miR-223-3p, and miR-155-5p expression and cardiac function (Figures 6A, C, D). This was an important finding because the correlation between miR-155-5p and cardiac function has been proposed to be involved in alterations in cross-bridge cycling and fibrosis. In contrast to miR-34a-3p, the expression levels of both miR-34a-5p (Figure 6B) and miR-34c-5p were also strongly correlated with the parameters of LV function. Furthermore, miR-34a-5p, unlike the other analysed miRNAs, showed clear expression differences between the control and treatment groups based on a cluster analysis. For miR-34a, expression of the 3′arm seems to correspond less to DAU-induced toxicity when compared to expression of the corresponding 5′arm.

**TABLE 3:** Correlation between miRNAs and cTnT, and between miRNAs and functional parameters of LV after the 10th weeks of DAU treatment.

	cTnT			LV dP/dt_max_			LV FS		
	R	*p*		R	*p*		R	*p*	
miR-**21**-3p	0.849	0.001	***	−0.761	0.01	**	−0.883	0.001	***
miR-**21**-5p	0.899	0.001	***	−0.89	0.001	***	−0.959	0.001	***
miR-**34a**-3p	0.561	0.05	*						
miR-**34a**-5p	0.746	0.001	***	−0.869	0.001	***	−0.779	0.001	***
miR-**34c**-5p	0.75	0.01	**	−0.948	0.001	***	−0.878	0.001	***
miR-**142**-3p	0.667	0.001	**	−0.659	0.05	*	−0.792	0.001	***
miR-**155**-5p	0.877	0.001	***	−0.851	0.001	***	−0.885	0.001	***
miR-**223**-3p	0.887	0.001	***	−0.803	0.001	***	−0.911	0.001	***
miR-**433**-3p	0.663	0.01	**				−0.75	0.01	**
miR-**504**-3p	−0.803	0.001	***	0.656	0.05	*	0.704	0.01	**
miR-**1298**-5p	0.714	0.01	**	−0.758	0.01	**	−0.738	0.01	**

### Targets of miRNAs

**FIGURE 7:**
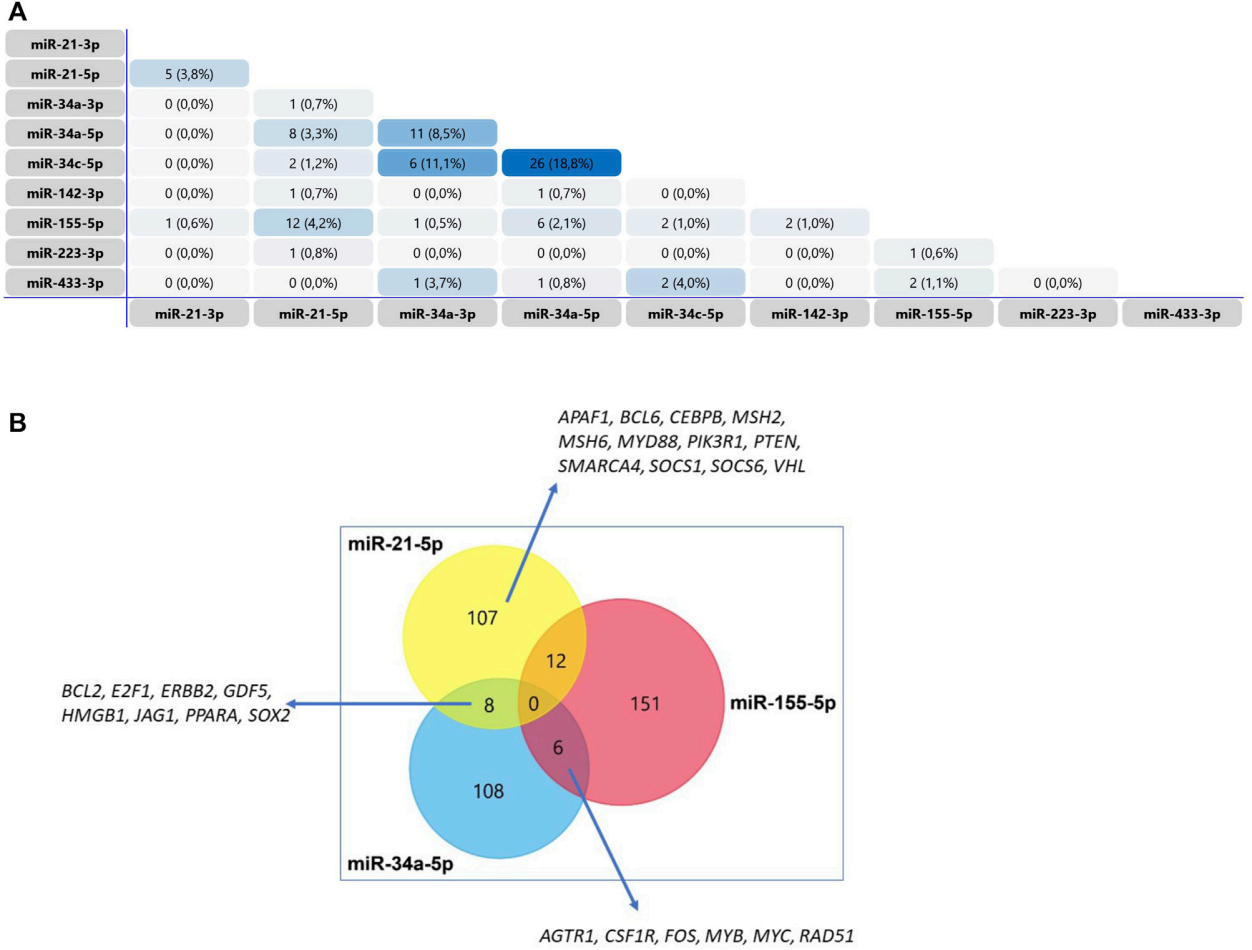
Venn diagram of the target genes of miRNAs with differential expression relative to the control in a model of chronic DAU-induced cardiomyopathy **(A)** The Venn diagram shows the number of genes shared between two selected miRNAs **(B)** Venn diagram of the target genes of miRNA-21-5p, miR-34a-5p and miR-155-5p, all of which were shown to be involved in the development of chronic DAU-induced cardiomyopathy in rabbits. The diagram shows the number of genes identified for each miRNA and the number of genes shared between the key miRNAs.

The Venn diagram presented in Figure 7A shows that most of the miRNAs that were found to be upregulated in the DAU-treated animals do not share the same target genes. The most important concordance was found between miRNA-21-5p and miR-34a-5p (BCL2, E2F1, ERBB2, GDF5, HMGB1, JAG1, PPARA, SOX2), miRNA 21-5p and miR-155-5p (APAF1, BCL6, CEBPB, MSH2, MSH6, MYD88, PIK3R1, PTEN, SMARCA4, SOCS1, SOCS6, VHL), and miR-34a-5p and miR-155-5p (AGTR1, CSF1R, FOS, MYB, MYC, RAD51) (Figure 7B). CCND1, TP53INP1 and NRF1 are verified targets of miR-504-3p, while CCND1 and Cx43 are proposed targets of miRNA-1298. The most common biological processes affected by miRNA-21-3p, miR-34a-5p, and miR-155-5p expression are related to gene transcription, apoptosis, DNA damage, DNA repair, and stress response. The biological processes in the myocardium that are affected by dysregulated miRNA expression are summarized in Table 4.

**TABLE 4:** Analysis of the main function of the deregulated miRNAs on our model of chronic daunorubicin-induced cardiomyopathy in rabbit.

Function	miRNA
DNA damage response	let-7f-2-3p, miR-20b-5p, miR-21-3p, miR-21-5p, miR-34a
Oxidative stress	miR-34a
Apoptosis	miR-21-3p, miR-21-5p, miR-34a
Autophagy	miR-21-3p, miR-21-5p, miR-34a
Calcium homeostasis	miR-20b-5p, miR-223
Abnormal vascular homeostasis	miR-21, miR-34, miR-155, miR-433, miR-1298
Inflammatory response	miR-21, miR-34
Fibrosis	miR-21, miR-34, miR-155, miR-433

### Western blotting of p53

**FIGURE 8:**
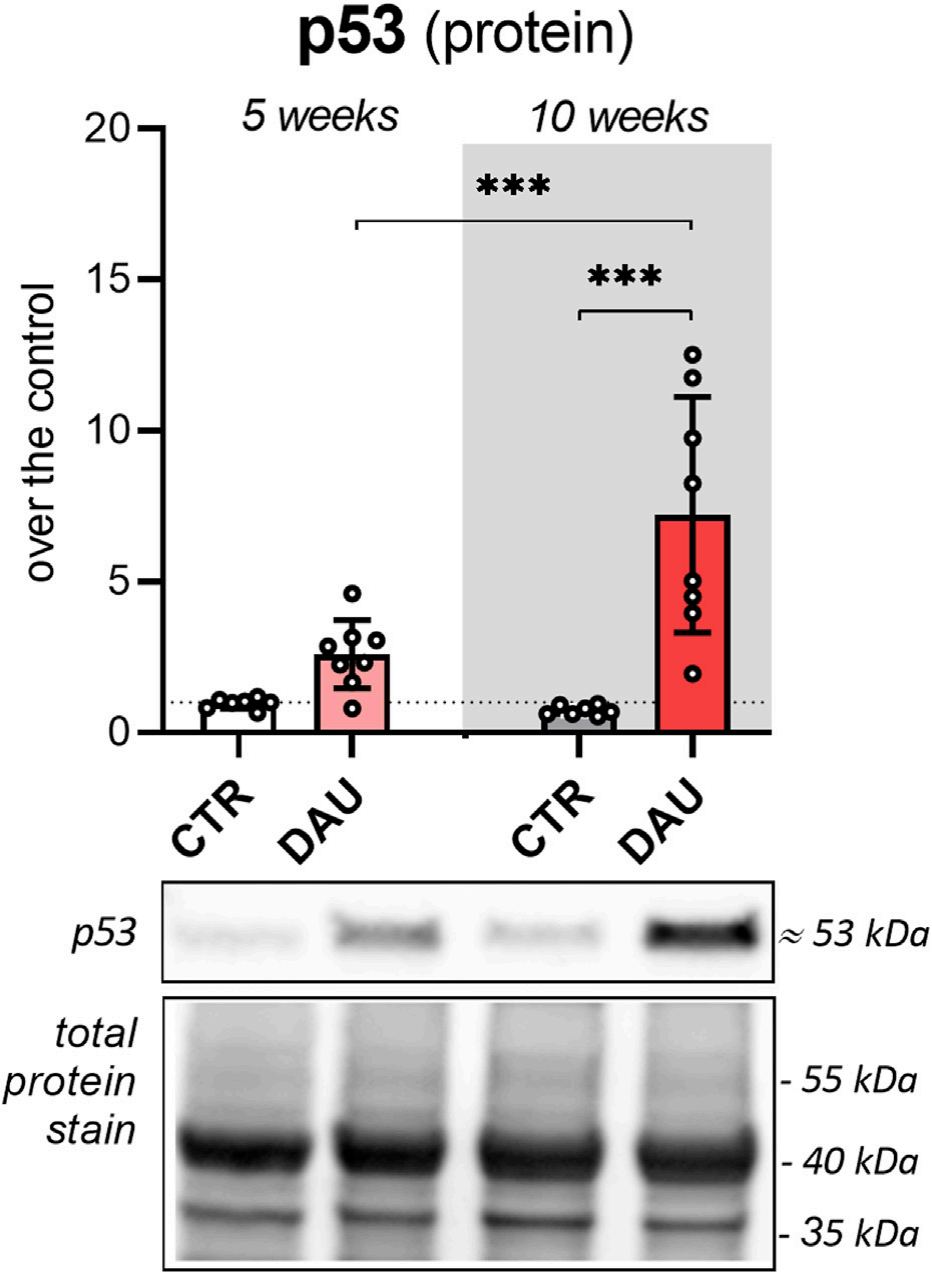
Analysis of p53 levels in the LV myocardium by Western blotting Myocardial p53 (molecular weight about 53 kDa) was analysed by WB, and total protein levels on the PVDF membrane were visualized by Stain-free imaging technology (Bio-Rad, Hercules, CA). Data are presented as the mean value ± SD. Significant changes are displayed as follows: *p* < 0.05 (*); *p* < 0.01 (**); *p* < 0.001 (***). 5 weeks corresponds to 5x DAU 3 mg/kg/week (cumulative dose ∼ 250 mg/m^2^); 10 weeks corresponds to 10x DAU 3 mg/kg/week (cumulative dose ∼ 500 mg/m^2^).

The observed levels of the protein p53, which is pivotal to the DNA damage response, showed insignificant differences between groups (control vs. DAU treatment) after 5 weeks of DAU treatment, with these differences becoming significant (*p* < 0.001) after 10 weeks of treatment. p53 levels significantly (*p* < 0.001) increased with the length of DAU treatment, i.e., from 5 weeks to 10 weeks of treatment (Figure 8; Supplementary Figure S1).

### Plasma miR-34a-5p concentrations

**FIGURE 9:**
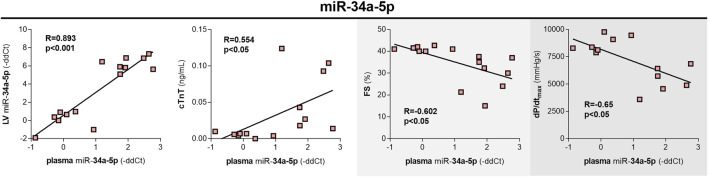
Plasma levels of miR-34a-5p, the correlations with expression in LV, and quantitative indices of toxic damage to the heart in rabbits after 10 weekly doses of DAU. miR-34a-5p expression in plasma vs. miR-34a-5p expression in the left ventricular myocardium. miR-34a-5p expression in plasma vs. plasma concentrations of cardiac troponin T, a marker of cardiac damage. miR-34a-5p expression in plasma vs. echocardiography-assessed left ventricular systolic function (FS - fractional shortening). miR-34a-5p expression in plasma vs. catheterization-assessed index of the left ventricular systolic function (dP/dtmax). The statistical significance of changes is displayed as follows: *p* < 0.05 (*); *p* < 0.01 (**); *p* < 0.001 (***).

In the plasma samples, only miR-34a-5p showed a significant (*p* < 0.001) increase after 10 weeks of treatment (4.15-fold change). Interestingly, the plasma levels of miR-34a-5p correlated with miR-34a-5p changes in the myocardium (R = 0.821, *p* < 0.001). The correlations between plasma levels of miR-34a-5p and cTnT (R = 0.634; *p* < 0.05), FS (R = −0.75; *p* < 0.001), and dP/dt_max_ (R = −0.61; *p* < 0.05) were weaker when compared to the correlations calculated based on myocardial expression, yet still significant (Figure 9).

## Discussion

The fact that miRNAs have strong and documented roles in the development of cardiovascular diseases (Wang, 2010; Romaine et al., 2015; Barwari et al., 2016; Chen et al., 2017; Dhingra and Vasan, 2017; Peters et al., 2020; Laggerbauer and Engelhardt, 2022; Gargiulo et al., 2023) means that it is expected that miRNAs are involved in the complex pathogenesis of ANT-induced cardiotoxicity. Notably, ANT exposure has already been reported to cause differential expression in various miRNAs (reviewed in Ruggeri et al., 2018b; Kawano and Adamcova, 2022; Yarmohammadi et al., 2023; Kuang et al., 2023; Chen and Xu, 2021; Li et al., 2022). Chaudhari et al. (2016) described the early deregulation of miR-187-3p, miR-182-5p, miR-486-3p, miR-486-5p, miR-34a-3p, miR-4423-3p, miR-34c-3p, miR-34c-5p, and miR-1303 in iPSCs exposed *in vitro* to 156 nM doxorubicin (DOX), as well as the prolonged upregulation of miR-182-5p, miR-4423-3p, and miR-34c-5p. Ruggeri et al. (2018a) determined that a cluster comprising miR-1-3p, miR-34a-5p, miR-133a-3p, and miR-499a-5p is associated with a differential cardiac response to DOX-induced cardiotoxicity in mice (6 × 4 mg/kg over 2 weeks and for weeks post-treatment FU). Nevertheless, more than half of the animals receiving the same treatment did not develop any cardiotoxicity over this study period, and the animals that developed cardiotoxicity mainly showed downregulation in the studied miRNAs (the only exception was an approximately 1.5-fold up-regulation of miR-34a-5p). In rats, DOX treatment (3 mg/kg for 4 weeks) resulted in the upregulation of 17 miRNAs (including miR-34c and miR-21, which were also found to be upregulated in our study, with relatively small changes, e.g., 2.25- and 3.21-fold, respectively). Moreover, DOX treatment was earlier reported to decrease the levels of 8 miRNAs, including let-7e, which did not show a significant change in our study (Vacchi-Suzzi et al., 2012). In another study that applied microarray transcriptional profiling, mice receiving i.v. DOX (3 mg/kg/week for 2–8 weeks) showed dysregulation in 24 miRNAs within the heart a week after the last dose was given (including miR-34a/b/c and miR-21-5p, both of which demonstrated changes in our study) (Desai et al., 2014). Only the results for miR-34a were validated through qPCR, with the assay revealing that this miRNA was upregulated 2.2- and 2.9-fold after six and 8 weeks of DOX treatment, respectively. In another rat model (six intraperitoneal injections of 2.5 mg/kg DOX over a period of 2 weeks), an approximately 1.7- and 2.5-fold increase in miR-34a expression was observed one and 4 weeks post-treatment (Piegari et al., 2016; Piegari et al., 2020).

To sum up, vast majority of studies of miRNA expression in ANT cardiotoxicity settings were done on mice and rats as the most common animals in cardiovascular research. However, mechanical and electrophysiological differences in cardiac function and different composition of contractile proteins may limit the translatability of the knowledge gained on rodent models to human medicine. The small heart size and fast heart rate are also other major disadvantages. In contrast to rodents, electrophysiological, mechanical and structural cardiac characteristics of rabbits resemble the human heart more closely, making them a suitable model for cardiac disease research (Hornyik et al., 2022).

In addition, it has been shown that chronic ANT cardiotoxicity induced in rabbits shares important histopathological, functional and biochemical hallmarks with the toxic damage described in humans and dexrazoxane provides significant cardioprotective effects herein similarly as in human medicine (Herman et al., 1981; Herman and Ferrans, 1986, Simunek et al.2 003). Hence, the present study takes the advantage of the study of changes in miRNA expression during ANT cardiotoxicity development in rabbits.

Alterations in miRNA expression in the LV myocardium during the course of ANT-induced cardiotoxicity development are still far from being fully understood. A primary knowledge gap is how changes in myocardial miRNA expression can be used to follow the development of cardiotoxicity from subclinical chronic cardiotoxicity to manifest cardiotoxicity with evident cardiomyopathy and HF. In prior empirical rodent studies, cardiac function and the translational biomarkers of cardiotoxicity were often not systematically studied; thus, the miRNA findings could not be correlated to the severity of cardiotoxicity. In the present study, we showed that multiple miRNAs in the LV myocardium demonstrate dysregulation after repeated ANT treatment prior to the onset of significant LV dysfunction (5 x DAU 3 mg/kg/week, cumulative dose 15 mg/kg, i.e., ∼ 250 mg/m^2^). Most of these changes were correlated with cTnT levels in individual animals, with cTnT a sensitive marker of myocardial microinjury. This implies that these particular changes were not accidental, but rather linked to the development of a cardiotoxic phenotype. In addition, animals that received an even greater cumulative dose of ANT (10 × 3 mg/kg/week, cumulative dose 30 mg/kg, ∼ 500 mg/m^2^), which results in a clear cardiotoxic phenotype, showed even greater dysregulation of these miRNAs. It is noteworthy that these changes also corresponded with the severity of LV dysfunction as determined by two independent approaches. This result provides further evidence that certain miRNAs are involved in the pathogenesis of chronic ANT-induced cardiotoxicity. In addition to miRNAs that showed dynamic expression changes as the cardiotoxic phenotype developed, certain miRNAs were only dysregulated in either the early or late periods of ANT cardiotoxicity development. Hence, these miRNAs may play a specific role in a given phase of cardiotoxicity development. This could be the case for miR-1298-5p, which showed the highest level of dysregulation in early periods of the experiment, with expression later decreasing.

Interestingly, one of the more prominent alterations found in both study intervals was related to the expression of miRNAs that are strongly associated with the DNA damage response (DDR) (Table 4). It has long been proposed that anthracycline cardiotoxicity is connected to direct oxidative stress induced via redox cycling of the drug molecule (Šimůnek et al., 2009; Štěrba et al., 2013), and that this oxidative damage can lead to DNA damage, which would trigger DDR (Yoshida et al., 2009). However, there is now increasing evidence that ANT-induced cardiotoxicity stems from specific interaction between ANTs and the beta isoform of TOP2 (TOP2B), which is the dominant isoform in terminally differentiated cardiomyocytes. The poisoning of this enzyme by ANTs leads to DNA double-strand breaks (Vejpongsa and Yeh, 2013). Zhang et al. (2012) found that the conditional knockout of TOP2B in cardiomyocytes prevents the development of chronic ANT-induced cardiotoxicity via prevention of DNA damage, p53-mediated DNA damage response (DDR), along with subsequent apoptotic signalling, and mitochondrial biogenesis impairment. Dexrazoxane, the only clinically effective cardioprotectant against ANT-induced cardiotoxicity, was recently found to act by preventing ANT-induced, TOP2B-dependent DNA damage in cardiomyocytes rather than via iron chelation/prevention of oxidative stress, which was the previously proposed mechanism (Jirkovský et al., 2021). In close DEX derivatives, *in vitro* and *in vivo* efficacy against ANT-induced cardiotoxicity is closely correlated to the ability to catalytically inhibit TOP2B and prevent the DNA damage resulting from an interaction between ANTs and TOP2B (Hasinoff et al., 2020; Jirkovská et al., 2021; Kollárova-Brázdova et al., 2021).

In our study, several of the miRNAs which showed significant changes in expression following DAU treatment are related to DDR. This is particularly relevant for members of the miR-34 family (Kawano and Adamcova, 2022; Li et al., 2022) as well as miR-504, but—to a certain extent—several others, including the miRNA-21 family, miR-20b-5p, miR-142-3p, and miR-155. Several of these latter miRNAs have been shown to target key mediators of DNA repair and replication in cancer cells, e.g., ATM, ATR, CHK1 and CHK2, DNA-PK, and WEE1 (Pilié et al., 2019), but evidence for these relationships in cardiomyocytes is mostly lacking. Concerning the miR-34 family, three members were found to be upregulated in both study intervals, with miR-34a-5p expression increasing almost 10-fold during the transition from subclinical phenotype to manifest cardiac damage. Moreover, the expression changes correlated well with cTnT levels, which are used as an early sign of cardiotoxicity, as well as markers of cardiac dysfunction at the later stages of the experiment.

The protein p53 directly stimulates the transcription of the miR-34 family, particularly miR-34a. The Western blotting experiments confirmed that p53 accumulates in the heart as ANT-induced cardiotoxicity shifts from subclinical to clinically manifest damage. It should be noted that miR-34a acts as a positive feedback regulator of p53 activity (Navarro and Lieberman, 2015). This effect is mainly mediated by inhibiting the translation of SIRT1, which normally keeps p53 deacetylated and prone to degradation by the ubiquitin proteasome system. A marked increase in miR-34 can thus stabilise p53 by preventing deacetylation, which will promote transcriptional activity (Solomon et al., 2006; Capaccia et al., 2022). Furthermore, decreased SIRT1 levels in the heart may have multiple other consequences, including the induction of apoptosis, cellular senescence, impairment of mitochondrial biogenesis, and mitochondrial damage with oxidative stress (Yamakuchi and Lowenstein, 2009; Hua et al., 2022). Another independent positive regulator of p53 function is miR-504-5p downregulation, which was seen in latter parts of the study, as this miRNA inhibits the transcription of p53 (Hu et al., 2010).

Several other *in vitro* studies have reported increased expression of miR-34 family members in the cardiomyocytes and heart tissue of different experimental models exposed to repeated cycles of ANT therapy (Desai et al., 2014; Zhu et al., 2017; Pellegrini et al., 2020; Piegari et al., 2020). In line with our observation, Desai et al. (2014), showed that miR-34 members are induced at early stages in the development of ANT-induced cardiotoxicity, prior to the onset of a cardiotoxic phenotype. Nevertheless, the relative increase in miR-34 expression was markedly lower than what we reported (2-3-fold) despite a significant cumulative dose of DOX in mice (15–24 mg/kg). It has been also shown that changes in miR-34 expression can be mitigated by the coadministration of a cardioprotective drug dexrazoxane (Zhu et al., 2017), which works by catalytically inhibiting TOP2B to prevent ANT-induced DNA damage in cardiomyocytes (Jirkovský et al., 2021). The detrimental role of miR-34a in ANT cardiotoxicity pathogenesis has been confirmed by miR-34a silencing (Piegari et al., 2020); more specifically, the silencing led to the de-repression of miR-34a target genes BCl2 and SIRT1 in the heart and significant amelioration of the cardiotoxic phenotype. This is in line with the effect of miR-34a silencing for several other cardiac injuries (Ooi et al., 2016; Hua et al., 2022). We found that plasma levels of miR-34-a at the end of the treatment period corresponded well with its myocardial levels; this suggests that miR-34-a could be used as a non-invasive biomarker of cardiac damage, although the sensitivity of this approach could be low for subclinical cardiotoxicity may be low, as there were no significant between-group differences in early phases of the experiment. Several other authors have reported the plasma miR-34a levels among patients treated with ANTs (Brown et al., 2022).

Several of the miRNAs that showed significantly increased expression in the heart after ANT treatment are important regulator of apoptosis. Members of the miR-34 family can induce apoptosis through positive feedback regulation of p53 signalling, which can result in the overexpression of proapoptotic factors (BAX, and PUMA, among others); furthermore, these miRNAs can directly repress the expression of antiapoptotic protein BCl-2, while SIRT1 deficiency can cause further pro-apoptotic signalling (Hua et al., 2022). miR-21 seems to have an anti-apoptotic role via targeting genes such as BTG2, PDCD4, PTEN, and FasL (Cheng and Zhang, 2010; Sayed et al., 2010; Surina et al., 2021). For instance, Tong et al. (2015) reported that miR-21 is upregulated in the myocardium of chronically DOX-treated mice and suggested that this alteration can prevent DOX-induced apoptosis by targeting BTG2. Data concerning the role of miR-142-3p in regulating cardiomyocyte apoptosis seems to be controversial (Wang et al., 2016; Zhan et al., 2017), with no data available from experiments on ANT-induced cardiotoxicity. MiR-155 has been reported to target antiapoptotic genes in various disease models, including a model of myocardial infarction (Guo et al., 2019), and increased expression of miR-155 after DOX treatment has been reported by other investigators (Xiao et al., 2022). To the best of our knowledge, the present study details—for the first time—strong upregulation of miR-1298 after both five and 10 weeks of ANT administration. According to Ouyang et al. (2022), miR-1298 overexpression reduces cardiomyocyte apoptosis in ischemia-reperfusion injury of the heart by inhibiting apoptosis (decreased BAX expression and increased Bcl-2 expression) and AMPK signalling activation.

Several of the miRNAs that were found to be dysregulated in the present study have been shown to be significantly involved in the regulation of oxidative stress and inflammation (Table 4), e.g., SIRT-1 inhibition induced by miR-34a expression can increase mitochondrial ROS generation via inhibition of PGC1a-regulated mitochondrial biogenesis and SOD2 expression. The latter events have been described by our group (Jirkovský et al., 2012), and have been documented in other models of chronic ANT cardiotoxicity (Zhang et al., 2012; Moulin et al., 2015); furthermore, these adverse effects have been shown to be preventable by DEX administration (Jirkovský et al., 2013) and TOP2B knock-out in cardiomyocytes (Zhang et al., 2012). MiR-21 (which also demonstrated a change in our study) has been reported to reduce SOD2 expression and induce oxidative stress (Tijsen et al., 2012). Both miR-34 and miR-21 have been linked to the inflammatory response, which is relevant to ANT-induced cardiotoxicity (Fabiani et al., 2021).

In addition to alterations in DDR-associated miRNA expression, we found expression changes in miRNAs typically related to cardiac remodelling, fibrosis, and dilated cardiomyopathy after the 10th administration of DAU. Pro-fibrotic miR-21, miR-34a, miR-155, and miR-433 increase the fibrosis-mediating ERK, p38, AKT, and SMAD or TGF-β1 signalling pathways (Adamcova et al., 2021). Most of these changes are described in the literature in models of other cardiac diseases than ANT-induced cardiotoxicity. It is necessary to note that with developing fibrosis, miRNAs are produced not only by cardiomyocytes but also by fibroblasts, leukocytes, and other cells of the myocardium. miR-433 plays a crucial role in regulating fibrosis (Tao et al., 2016), with JNK1 and AZIN1 proposed as the target genes that mediate the fibrotic effect (Ooi et al., 2016). It is important to note that MiR-223 is considered to be a marker of heart failure (Zhang et al., 2021). Furthermore, miR-223-3p directly inhibits troponin I3 interacting kinase (TNNI3K), which decreases the phosphorylation of cardiac troponin I, a downstream target of TNNI3K. This can reverse the prior increase in intracellular Ca^2+^ concentrations and contractility of cardiomyocytes (Wang et al., 2015). In addition, Voellenkle et al. (2010) previously described changes in miRNA 142-3p expression among patients with non-ischemic dilated cardiomyopathy.

Following the fifth administration of DAU, only two miRNAs were found to be upregulated, i.e., miR-let-7f-2-3p and miR-20b. Previously published literature has reported that miR-let-7f-2-3p aggravates DOX-induced cardiotoxicity by inhibiting XPO1-mediated HAX-1 nuclear export, which increases apoptosis (Grzybowska et al., 2013; Bidwell et al., 2018; Guo et al., 2018; Liu et al., 2019). HAX-1 is also a critical gatekeeper of SR calcium cycling and contractility in the heart (Bidwell et al., 2018). miR-20b targets mitofusin 2 (Mfn2), which is located in the outer mitochondrial membrane, and this inhibition leads to the dysregulation of mitochondrial Ca^2+^ buffering capacity (Qiu et al., 2020).

The most significant changes in miRNA expression found through our model of ANT-induced cardiotoxicity were for miRNA-34a and miRNA-1298. We can only speculate about the role of miRNA-1298 in the pathogenesis of ANT-induced cardiotoxicity. There is the potential that this miRNA can exert a cardioprotective effect to counteract the detrimental miR-34-p53 pathway related to DDR. MiRNA-1298 improves cardiomyocyte regeneration via cyclin D1. Haybar et al. (2021) even suggest using cyclin D1 as a marker for following the effects of regenerative treatments against chemotherapy-induced cardiotoxicity. However, these hypotheses need further investigation.

## Conclusion

Our study provides new insights into miRNA expression in the left ventricle during the development of ANT-induced cardiotoxicity using a well-validated model of chronic ANT cardiomyopathy. Across the two distinct phases of the study (subclinical and fully developed cardiomyopathy), the significant changes were found in thirteen examined miRNAs, which are known to be mainly related to DNA damage and DDR signalling, apoptosis regulation, cardiac remodelling, fibrosis, and oxidative stress. Thus, our findings fit the current view on the pathophysiology of ANT cardiotoxicity and suggest that the miRNAs play a crucial role in this process. To the best of our knowledge, this is the first study utilizing other than rodent species to document ANT-induced changes in miRNA expression in the left ventricular myocardium at different stages of cardiotoxicity development. Furthermore, the present study found that most of these changes correspond well with markers of the toxic damage (cTnT, FS, dp/dt_max_), which was unknown so far. In addition, the correlation of the plasma levels of miR-34a-5p with its myocardial expression suggests that a non-invasive approach to analyse such miRNAs can be a feasible and valuable tool for cardio-oncology. In the future, miRNAs could play a role in the diagnosis and estimation of prognosis and as a therapeutic target of chemotherapy-induced cardiotoxicity. MiRNAs have significant potential for preventing, alleviating, and restoring cardiac dysfunction and adverse ventricular remodelling.

## Limitations of the study

The main limitation of the study is that the role of some miRNAs in the development of cardiotoxicity has not been completely understood. For example, some interesting findings, like the dramatic increase in miR-1298-5p expression in the subclinical stage, deserve further research and attention. Gene disruption or antagomir/agomir administration experiments might be useful to clarify the functional role of such miRNAs in ANT-induced cardiotoxicity. In addition, our study was designated as an exploratory analysis, which should be followed by the precise validation of results with larger groups of animals. Attention should also be paid to other representatives of the ANT class to determine whether all the observed miRNA changes can be regarded as class effects attributable to ANT cardiotoxicity. Concerning the potential importance of sexual dimorphism, a similar study should be also done on female animals. Unlike the studies performed on rodents, the physiological properties of a rabbit’s myocardium are closer to the human heart in several aspects. However, the translational potential of our research should be confirmed on other non-rodent animals such as pigs and monkeys. Addressing these limitations is warranted in further investigative studies.

## Data Availability

The original contributions presented in the study are included in the article/Supplementary Material, further inquiries can be directed to the corresponding author.

## References

[B1] Adamcová M. Geršl V. Hrdina R. Mělka M. Mazurová Y. Vávrová J. (1999). Cardiac troponin T as a marker of myocardial damage caused by antineoplastic drugs in rabbits. J. Cancer Res. Clin. Oncol. 125 (5), 268–274. 10.1007/s004320050273 10359131 PMC12199899

[B2] Adamcova M. Kawano I. Simko F. (2021). The impact of microRNAs in renin–angiotensin-system-induced cardiac remodelling. Int. J. Mol. Sci. 22, 4762. 10.3390/ijms22094762 33946230 PMC8124994

[B3] Adamcova M. Lencova-Popelova O. Jirkovsky E. Mazurova Y. Palicka V. Simko F. (2015). Experimental determination of diagnostic window of cardiac troponins in the development of chronic anthracycline cardiotoxicity and estimation of its predictive value. Int. J. Cardiol. 15 (201), 358–367. 10.1016/j.ijcard.2015.07.103 26310978

[B4] Alves M. T. da Conceição I. M. C. A. de Oliveira A. N. Oliveira H. H. M. Soares C. E. de Paula Sabino A. (2022). microRNA miR-133a as a biomarker for doxorubicin-induced cardiotoxicity in women with breast cancer: a signaling pathway investigation. Cardiovasc Toxicol. 22, 655–662. 10.1007/s12012-022-09748-4 35524907

[B5] Bartel D. P. (2004). MicroRNAs: genomics, biogenesis, mechanism, and function. Cell 116, 281–297. 10.1016/S0092-8674(04)00045-5 14744438

[B6] Barwari T. Joshi A. Mayr M. (2016). MicroRNAs in cardiovascular disease. J. Am. Coll. Cardiol. 68, 2577–2584. 10.1016/j.jacc.2016.09.945 27931616

[B7] Bidwell P. A. Haghighi K. Kranias E. G. (2018). The antiapoptotic protein HAX-1 mediates half of phospholamban’s inhibitory activity on calcium cycling and contractility in the heart. J. Biol. Chem. 293, 359–367. 10.1074/jbc.RA117.000128 29150445 PMC5766917

[B8] Brown C. Mantzaris M. Nicolaou E. Karanasiou G. Papageorgiou E. Curigliano G. (2022). A systematic review of miRNAs as biomarkers for chemotherapy-induced cardiotoxicity in breast cancer patients reveals potentially clinically informative panels as well as key challenges in miRNA research. Cardio-Oncology 8, 16. 10.1186/s40959-022-00142-1 36071532 PMC9450324

[B9] Capaccia C. Diverio S. Zampini D. Guelfi G. (2022). The complex interaction between P53 and miRNAs joins new awareness in physiological stress responses. Cells 11, 1631. 10.3390/cells11101631 35626668 PMC9139524

[B10] Chaudhari U. Nemade H. Gaspar J. A. Hescheler J. Hengstler J. G. Sachinidis A. (2016). MicroRNAs as early toxicity signatures of doxorubicin in human-induced pluripotent stem cell-derived cardiomyocytes. Arch. Toxicol. 90, 3087–3098. 10.1007/s00204-016-1668-0 26842497 PMC5104806

[B11] Chen C. Ponnusamy M. Liu C. Gao J. Wang K. Li P. (2017). MicroRNA as a therapeutic target in cardiac remodeling. Biomed. Res. Int. 2017, 1278436. 10.1155/2017/1278436 29094041 PMC5637866

[B12] Chen L. Xu Y. (2021). MicroRNAs as biomarkers and therapeutic targets in doxorubicin-induced cardiomyopathy: a review. Front. Cardiovasc Med. 8, 740515. 10.3389/fcvm.2021.740515 34901206 PMC8653425

[B13] Chen Y. Xu Y. Deng Z. Wang Y. Zheng Y. Jiang W. (2021). MicroRNA expression profiling involved in doxorubicin-induced cardiotoxicity using high-throughput deep-sequencing analysis. Oncol. Lett. 22, 560. 10.3892/ol.2021.12821 34093775 PMC8170198

[B14] Cheng Y. Zhang C. (2010). MicroRNA-21 in cardiovascular disease. J. Cardiovasc Transl. Res. 3, 251–255. 10.1007/s12265-010-9169-7 20560046 PMC3611957

[B15] Chistiakov D. A. Orekhov A. N. Bobryshev Y. V. (2016). Cardiac-specific miRNA in cardiogenesis, heart function, and cardiac pathology (with focus on myocardial infarction). J. Mol. Cell Cardiol. 94, 107–121. 10.1016/j.yjmcc.2016.03.015 27056419

[B16] Choo K. B. Soon Y. L. Nguyen P. N. N. Hiew M. S. Y. Huang C.-J. (2014). MicroRNA-5p and -3p co-expression and cross-targeting in colon cancer cells. J. Biomed. Sci. 21, 95. 10.1186/s12929-014-0095-x 25287248 PMC4195866

[B17] Desai V. G. Kwekel C. Vijay V. Moland C. L. Herman E. H. Lee T. (2014). Early biomarkers of doxorubicin-induced heart injury in a mouse model. Toxicol. Appl. Pharmacol. 281, 221–229. 10.1016/j.taap.2014.10.006 25448438

[B18] Dhingra R. Vasan R. S. (2017). Biomarkers in cardiovascular disease: statistical assessment and section on key novel heart failure biomarkers. Trends Cardiovasc Med. 27, 123–133. 10.1016/j.tcm.2016.07.005 27576060 PMC5253084

[B19] Ewer M. S. Ewer S. M. (2015). Erratum: cardiotoxicity of anticancer treatments. Nat. Rev. Cardiol. 12, 620. 10.1038/nrcardio.2015.133 26292190

[B20] Fabiani I. Aimo A. Grigoratos C. Castiglione V. Gentile F. Saccaro L. F. (2021). Oxidative stress and inflammation: determinants of anthracycline cardiotoxicity and possible therapeutic targets. Heart Fail Rev. 26 (4), 881–890. 10.1007/s10741-020-10063-9 33319255 PMC8149360

[B21] Gargiulo P. Marzano F. Salvatore M. Basile C. Buonocore D. Parlati A. L. M. (2023). MicroRNAs: diagnostic, prognostic and therapeutic role in heart failure—a review. Esc. Heart Fail 10, 753–761. 10.1002/ehf2.14153 36349485 PMC10053166

[B22] Grzybowska E. A. Zayat V. Konopiński R. Trębińska A. Szwarc M. Sarnowska E. (2013). HAX-1 is a nucleocytoplasmic shuttling protein with a possible role in mRNA processing. FEBS J. 280, 256–272. 10.1111/febs.12066 23164465

[B23] Guo J. Liu H.-B. Sun C. Yan X.-Q. Hu J. Yu J. (2019). MicroRNA-155 promotes myocardial infarction-induced apoptosis by targeting RNA-binding protein QKI. Oxid. Med. Cell Longev. 2019, 4579806–4579814. 10.1155/2019/4579806 31191799 PMC6525929

[B24] Guo X.-B. Deng X. Wei Y. (2018). Hematopoietic substrate-1-associated protein X-1 regulates the proliferation and apoptosis of endothelial progenitor cells through akt pathway modulation. Stem Cells 36, 406–419. 10.1002/stem.2741 29139175

[B25] Ha T.-Y. (2011). MicroRNAs in human diseases: from cancer to cardiovascular disease. Immune Netw. 11, 135–154. 10.4110/in.2011.11.3.135 21860607 PMC3153666

[B26] Harada M. Luo X. Murohara T. Yang B. Dobrev D. Nattel S. (2014). MicroRNA regulation and cardiac calcium signaling: role in cardiac disease and therapeutic potential. Circ. Res. 114, 689–705. 10.1161/CIRCRESAHA.114.301798 24526675

[B27] Hasinoff B. B. Patel D. Wu X. (2020). The role of topoisomerase IIβ in the mechanisms of action of the doxorubicin cardioprotective agent dexrazoxane. Cardiovasc Toxicol. 20, 312–320. 10.1007/s12012-019-09554-5 31773441

[B28] Haybar H. Shahrouzian M. Gatavizadeh Z. Saki N. Maniati M. Zayeri Z. D. (2021). Cyclin D1: a golden gene in cancer, cardiotoxicity, and cardioprotection *jundishapur J chronic dis Care* . Jundishapur J. Chronic Dis. Care 10 (3), e112413. 10.5812/jjcdc.112413

[B29] Herman E. H. Ferrans V. J. (1986). Pretreatment with ICRF-187 provides long-lasting protection against chronic daunorubicin cardiotoxicity in rabbits. Cancer Chemother. Pharmacol. 16 (2), 102–106. 10.1007/BF00256157 3081268

[B30] Herman E. H. Ferrans V. J. Jordan W. Ardalan B. (1981). Reduction of chronic daunorubicin cardiotoxicity by ICRF-187 in rabbits. Res. Commun. Chem. Pathol. Pharmacol. 31 (1), 85–97. 6789416

[B31] Holmgren G. Synnergren J. Andersson C. X. Lindahl A. Sartipy P. (2016). MicroRNAs as potential biomarkers for doxorubicin-induced cardiotoxicity. Toxicol. Vitro 34, 26–34. 10.1016/j.tiv.2016.03.009 27033315

[B32] Hornyik T. Rieder M. Castiglione A. Major P. Baczko I. Brunner M. (2022). Transgenic rabbit models for cardiac disease research. Br. J. Pharmacol. 179 (5), 938–957. 10.1111/bph.15484 33822374

[B33] Hu W. Chan C. S. Wu R. Zhang C. Sun Y. Song J. S. (2010). Negative regulation of tumor suppressor p53 by MicroRNA miR-504. Mol. Cell 38, 689–699. 10.1016/j.molcel.2010.05.027 20542001 PMC2900922

[B34] Hua C.-C. Liu X.-M. Liang L.-R. Wang L.-F. Zhong J.-C. (2022). Targeting the microRNA-34a as a novel therapeutic strategy for cardiovascular diseases. Front. Cardiovasc Med. 8, 784044. 10.3389/fcvm.2021.784044 35155600 PMC8828972

[B35] Ilieva M. Panella R. Uchida S. (2022). MicroRNAs in cancer and cardiovascular disease. Cells 11, 3551. 10.3390/cells11223551 36428980 PMC9688578

[B36] Jirkovská A. Karabanovich G. Kubeš J. Skalická V. Melnikova I. Korábečný J. (2021). Structure–activity relationship study of dexrazoxane analogues reveals ICRF-193 as the most potent bisdioxopiperazine against anthracycline toxicity to cardiomyocytes due to its strong topoisomerase IIβ interactions. J. Med. Chem. 64, 3997–4019. 10.1021/acs.jmedchem.0c02157 33750129

[B37] Jirkovský E. Jirkovská A. Bavlovič-Piskáčková H. Skalická V. Pokorná Z. Karabanovich G. (2021). Clinically translatable prevention of anthracycline cardiotoxicity by dexrazoxane is mediated by topoisomerase II beta and not metal chelation. Circ. Heart Fail 14, e008209. 10.1161/CIRCHEARTFAILURE.120.008209 34551586

[B38] Jirkovský E. Lenčová-Popelová O. Hroch M. Adamcová M. Mazurová Y. Vávrová J. (2013). Early and delayed cardioprotective intervention with dexrazoxane each show different potential for prevention of chronic anthracycline cardiotoxicity in rabbits. Toxicology 311, 191–204. 10.1016/j.tox.2013.06.012 23831762

[B39] Jirkovský E. Popelová O. Křiváková-Staňková P. Vávrová A. Hroch M. Hašková P. (2012). Chronic anthracycline cardiotoxicity: molecular and functional analysis with focus on nuclear factor erythroid 2-related factor 2 and mitochondrial biogenesis pathways. J. Pharmacol. Exp. Ther. 343, 468–478. 10.1124/jpet.112.198358 22915767

[B40] Kawano I. Adamcova M. (2022). MicroRNAs in doxorubicin-induced cardiotoxicity: the DNA damage response. Front. Pharmacol. 13, 1055911. 10.3389/fphar.2022.1055911 36479202 PMC9720152

[B41] Kern F. Aparicio-Puerta E. Li Y. Fehlmann T. Kehl T. Wagner V. (2021). miRTargetLink 2.0—interactive miRNA target gene and target pathway networks. Nucleic Acids Res. 49, W409–W416. 10.1093/nar/gkab297 34009375 PMC8262750

[B42] Kollárová-Brázdová P. Lenčová-Popelová O. Karabanovich G. Kocúrová-Lengvarská J. Kubeš J. Váňová N. (2021). Prodrug of ICRF-193 provides promising protective effects against chronic anthracycline cardiotoxicity in a rabbit model *in vivo* . Clin. Sci. 135, 1897–1914. 10.1042/CS20210311 34318878

[B43] Kuang Z. Wu J. Tan Y. Zhu G. Li J. Wu M. (2023). MicroRNA in the diagnosis and treatment of doxorubicin-induced cardiotoxicity. Biomolecules 13, 568. 10.3390/biom13030568 36979503 PMC10046787

[B44] Laggerbauer B. Engelhardt S. (2022). MicroRNAs as therapeutic targets in cardiovascular disease. J. Clin. Investigation 132, e159179. 10.1172/JCI159179 PMC9151703 35642640

[B45] Li H. Zhan J. Chen C. Wang D. (2022). MicroRNAs in cardiovascular diseases. Med. Rev. 2, 140–168. 10.1515/mr-2021-0001 PMC10471109 37724243

[B46] Liu Y. Duan C. Liu W. Chen X. Wang Y. Liu X. (2019). Upregulation of let-7f-2-3p by long noncoding RNA NEAT1 inhibits XPO1-mediated HAX-1 nuclear export in both *in vitro* and *in vivo* rodent models of doxorubicin-induced cardiotoxicity. Arch. Toxicol. 93, 3261–3276. 10.1007/s00204-019-02586-4 31570982

[B47] Moulin M. Piquereau J. Mateo P. Fortin D. Rucker-Martin C. Gressette M. (2015). Sexual dimorphism of doxorubicin-mediated cardiotoxicity: potential role of energy metabolism remodeling. Circ. Heart Fail 8, 98–108. 10.1161/CIRCHEARTFAILURE.114.001180 25420486

[B48] Navarro F. Lieberman J. (2015). miR-34 and p53: new insights into a complex functional relationship. PLoS One 10, e0132767. 10.1371/journal.pone.0132767 26177460 PMC4503669

[B49] Nishimura Y. Kondo C. Morikawa Y. Tonomura Y. Torii M. Yamate J. (2015). Plasma miR-208 as a useful biomarker for drug-induced cardiotoxicity in rats. J. Appl. Toxicol. 35, 173–180. 10.1002/jat.3044 25092230

[B50] O’Brien J. Hayder H. Zayed Y. Peng C. (2018). Overview of MicroRNA biogenesis, mechanisms of actions, and circulation. Front. Endocrinol. (Lausanne) 9, 402. 10.3389/fendo.2018.00402 30123182 PMC6085463

[B51] Oliveira-Carvalho V. Ferreira L. R. P. Bocchi E. A. (2015). Circulating mir-208a fails as a biomarker of doxorubicin-induced cardiotoxicity in breast cancer patients. J. Appl. Toxicol. 35, 1071–1072. 10.1002/jat.3185 26046768

[B52] Ooi J. Y. Y. Bernardo B. C. McMullen J. R. (2016). Therapeutic potential of targeting microRNAs to regulate cardiac fibrosis: miR-433 a new fibrotic player. Ann. Transl. Med. 4, 548. 10.21037/atm.2016.12.01 28149909 PMC5233506

[B53] Ouyang C. Huang L. Ye X. Ren M. Han Z. (2022). Overexpression of miR-1298 attenuates myocardial ischemia–reperfusion injury by targeting PP2A. J. Thromb. Thrombolysis 53, 136–148. 10.1007/s11239-021-02540-1 34351558

[B54] Pellegrini L. Sileno S. D’Agostino M. Foglio E. Florio M. C. Guzzanti V. (2020). MicroRNAs in cancer treatment-induced cardiotoxicity. Cancers (Basel) 12, 704. 10.3390/cancers12030704 32192047 PMC7140035

[B55] Pereira J. D. Tosatti J. A. G. Simões R. Luizon M. R. Gomes K. B. Alves M. T. (2020). microRNAs associated to anthracycline-induced cardiotoxicity in women with breast cancer: a systematic review and pathway analysis. Biomed. Pharmacother. 131, 110709. 10.1016/j.biopha.2020.110709 32937248

[B56] Peters L. J. F. Biessen E. A. L. Hohl M. Weber C. van der Vorst E. P. C. Santovito D. (2020). Small things matter: relevance of MicroRNAs in cardiovascular disease. Front. Physiol. 11, 793. 10.3389/fphys.2020.00793 32733281 PMC7358539

[B57] Piegari E. Cozzolino A. Ciuffreda L. P. Cappetta D. De Angelis A. Urbanek K. (2020). Cardioprotective effects of miR-34a silencing in a rat model of doxorubicin toxicity. Sci. Rep. 10, 12250. 10.1038/s41598-020-69038-3 32704131 PMC7378226

[B58] Piegari E. Russo R. Cappetta D. Esposito G. Urbanek K. Dell’Aversana C. (2016). MicroRNA-34a regulates doxorubicin-induced cardiotoxicity in rat. Oncotarget 7, 62312–62326. 10.18632/oncotarget.11468 27694688 PMC5308729

[B59] Pilié P. G. Tang C. Mills G. B. Yap T. A. (2019). State-of-the-art strategies for targeting the DNA damage response in cancer. Nat. Rev. Clin. Oncol. 16, 81–104. 10.1038/s41571-018-0114-z 30356138 PMC8327299

[B60] Qiu Y. Cheng R. Liang C. Yao Y. Zhang W. Zhang J. (2020). MicroRNA-20b promotes cardiac hypertrophy by the inhibition of mitofusin 2-mediated inter-organelle Ca2+ cross-talk. Mol. Ther. Nucleic Acids 19, 1343–1356. 10.1016/j.omtn.2020.01.017 32160705 PMC7036712

[B61] Renu K. Abilash V. G. Tirupathi Pichiah P. B. Arunachalam S. (2018). Molecular mechanism of doxorubicin-induced cardiomyopathy – an update. Eur. J. Pharmacol. 818, 241–253. 10.1016/j.ejphar.2017.10.043 29074412

[B62] Rigaud V. O.-C. Ferreira L. R. P. Ayub-Ferreira S. M. Ávila M. S. Brandão S. M. G. Cruz F. D. (2017). Circulating miR-1 as a potential biomarker of doxorubicin-induced cardiotoxicity in breast cancer patients. Oncotarget 8, 6994–7002. 10.18632/oncotarget.14355 28052002 PMC5351685

[B63] Romaine S. P. R. Tomaszewski M. Condorelli G. Samani N. J. (2015). MicroRNAs in cardiovascular disease: an introduction for clinicians. Heart 101, 921–928. 10.1136/heartjnl-2013-305402 25814653 PMC4484262

[B64] Ruggeri C. Gioffré S. Achilli F. Colombo G. I. D’Alessandra Y. (2018a). Role of microRNAs in doxorubicin-induced cardiotoxicity: an overview of preclinical models and cancer patients. Heart Fail Rev. 23, 109–122. 10.1007/s10741-017-9653-0 28944400 PMC5756562

[B65] Ruggeri C. Gioffré S. Chiesa M. Buzzetti M. Milano G. Scopece A. (2018b). A specific circulating MicroRNA cluster is associated to late differential cardiac response to doxorubicin-induced cardiotoxicity *in vivo* . Dis. Markers 2018, 8395651–8395659. 10.1155/2018/8395651 30627229 PMC6304816

[B66] Sawicki K. T. Sala V. Prever L. Hirsch E. Ardehali H. Ghigo A. (2021). Preventing and treating anthracycline cardiotoxicity: new insights. Annu. Rev. Pharmacol. Toxicol. 61, 309–332. 10.1146/annurev-pharmtox-030620-104842 33022184

[B67] Sayed D. He M. Hong C. Gao S. Rane S. Yang Z. (2010). MicroRNA-21 is a downstream effector of AKT that mediates its antiapoptotic effects via suppression of fas ligand. J. Biol. Chem. 285, 20281–20290. 10.1074/jbc.M110.109207 20404348 PMC2888441

[B68] Šimůnek T. Klimtová I. Adamcová M. Geršl V. Hrdina R. Štěrba M. (2003). Cardiac troponin T as an indicator of reduced left ventricular contractility in experimental anthracycline-induced cardiomyopathy. Cancer Chemother. Pharmacol. 52, 43–434. 10.1007/s00280-003-0675-z 14618274

[B69] Šimůnek T. Štěrba M. Popelová O. Adamcová M. Hrdina R. Geršl V. (2009). Anthracycline-induced cardiotoxicity: overview of studies examining the roles of oxidative stress and free cellular iron. Pharmacol. Rep. 61, 154–171. 10.1016/S1734-1140(09)70018-0 19307704

[B70] Solomon J. M. Pasupuleti R. Xu L. McDonagh T. Curtis R. DiStefano P. S. (2006). Inhibition of SIRT1 catalytic activity increases p53 acetylation but does not alter cell survival following DNA damage. Mol. Cell Biol. 26, 28–38. 10.1128/MCB.26.1.28-38.2006 16354677 PMC1317617

[B71] Štěrba M. Popelová O. Vávrová A. Jirkovský E. Kovaříková P. Geršl V. (2013). Oxidative stress, redox signaling, and metal chelation in anthracycline cardiotoxicity and pharmacological cardioprotection. Antioxid. Redox Signal 18, 899–929. 10.1089/ars.2012.4795 22794198 PMC3557437

[B72] Sterba M. Simůnek T. Popelová O. Potácová A. Adamcová M. Mazurová Y. (2007). Early detection of anthracycline cardiotoxicity in a rabbit model: left ventricle filling pattern versus troponin T determination. Physiol. Res. 56 (5), 535–545. 10.33549/physiolres.931025 17184149

[B73] Surina S. Fontanella R. A. Scisciola L. Marfella R. Paolisso G. Barbieri M. (2021). miR-21 in human cardiomyopathies. Front. Cardiovasc Med. 8, 767064. 10.3389/fcvm.2021.767064 34778418 PMC8578278

[B74] Tao L. Bei Y. Chen P. Lei Z. Fu S. Zhang H. (2016). Crucial role of miR-433 in regulating cardiac fibrosis. Theranostics 6, 2068–2083. 10.7150/thno.15007 27698941 PMC5039681

[B75] Thum T. Galuppo P. Wolf C. Fiedler J. Kneitz S. van Laake L. W. (2007). MicroRNAs in the human heart: a clue to fetal gene reprogramming in heart failure. Circulation 116, 258–267. 10.1161/CIRCULATIONAHA.107.687947 17606841

[B76] Tijsen A. J. Pinto Y. M. Creemers E. E. (2012). Non-cardiomyocyte microRNAs in heart failure. Cardiovasc Res. 93, 573–582. 10.1093/cvr/cvr344 22180601

[B77] Tong Z. Jiang B. Wu Y. Liu Y. Li Y. Gao M. (2015). MiR-21 protected cardiomyocytes against doxorubicin-induced apoptosis by targeting BTG2. Int. J. Mol. Sci. 16, 14511–14525. 10.3390/ijms160714511 26132560 PMC4519855

[B78] Vacchi-Suzzi C. Bauer Y. Berridge B. R. Bongiovanni S. Gerrish K. Hamadeh H. K. (2012). Perturbation of microRNAs in rat heart during chronic doxorubicin treatment. PLoS One 7, e40395. 10.1371/journal.pone.0040395 22859947 PMC3409211

[B79] Vejpongsa P. Yeh E. T. H. (2013). Topoisomerase 2β: a promising molecular target for primary prevention of anthracycline-induced cardiotoxicity. Clin. Pharmacol. Ther. 95, 45–52. 10.1038/clpt.2013.201 24091715

[B80] Voellenkle C. van Rooij J. Cappuzzello C. Greco S. Arcelli D. Di Vito L. (2010). MicroRNA signatures in peripheral blood mononuclear cells of chronic heart failure patients. Physiol. Genomics 42, 420–426. 10.1152/physiolgenomics.00211.2009 20484156

[B81] Wang Y. Ouyang M. Wang Q. Jian Z. (2016). MicroRNA-142-3p inhibits hypoxia/reoxygenation-induced apoptosis and fibrosis of cardiomyocytes by targeting high mobility group box 1. Int. J. Mol. Med. 38, 1377–1386. 10.3892/ijmm.2016.2756 28025989 PMC5065300

[B82] Wang Y.-S. Zhou J. Hong K. Cheng X.-S. Li Y.-G. (2015). MicroRNA-223 displays a protective role against cardiomyocyte hypertrophy by targeting cardiac troponin I-interacting kinase. Cell. Physiology Biochem. 35, 1546–1556. 10.1159/000373970 25792377

[B83] Wang Z. (2010). MicroRNAs and cardiovascular disease. *Bentham e-Book.* SBN: 978-1-60805-184-7. 10.2174/97816080518471100101

[B84] Xiao Z. Wei S. Huang J. Liu J. Liu J. Zhang B. (2022). Noncoding RNA-associated competing endogenous RNA networks in doxorubicin-induced cardiotoxicity. DNA Cell Biol. 41, 657–670. 10.1089/dna.2022.0022 35593913

[B85] Yamakuchi M. Lowenstein C. J. (2009). MiR-34, SIRT1, and p53: the feedback loop. Cell Cycle 8, 712–715. 10.4161/cc.8.5.7753 19221490

[B86] Yarmohammadi F. Ebrahimian Z. Karimi G. (2023). MicroRNAs target the PI3K/Akt/p53 and the Sirt1/Nrf2 signaling pathways in doxorubicin‐induced cardiotoxicity. J. Biochem. Mol. Toxicol. 37, e23261. 10.1002/jbt.23261 36416353

[B87] Yoshida M. Shiojima I. Ikeda H. Komuro I. (2009). Chronic doxorubicin cardiotoxicity is mediated by oxidative DNA damage-ATM-p53-apoptosis pathway and attenuated by pitavastatin through the inhibition of Rac1 activity. J. Mol. Cell Cardiol. 47, 698–705. 10.1016/j.yjmcc.2009.07.024 19660469

[B88] Zhan L. Lei S. Li W. Zhang Y. Wang H. Shi Y. (2017). Suppression of microRNA-142-5p attenuates hypoxia-induced apoptosis through targeting SIRT7. Biomed. Pharmacother. 94, 394–401. 10.1016/j.biopha.2017.07.083 28772218

[B89] Zhang M.-W. Shen Y.-J. Shi J. Yu J.-G. (2021). MiR-223-3p in cardiovascular diseases: a biomarker and potential therapeutic target. Front. Cardiovasc Med. 7, 610561. 10.3389/fcvm.2020.610561 33553260 PMC7854547

[B90] Zhang S. Liu X. Bawa-Khalfe T. Lu L.-S. Lyu Y. L. Liu L. F. (2012). Identification of the molecular basis of doxorubicin-induced cardiotoxicity. Nat. Med. 18, 1639–1642. 10.1038/nm.2919 23104132

[B91] Zhu J.-N. Fu Y.-H. Hu Z. Li W.-Y. Tang C.-M. Fei H.-W. (2017). Activation of miR-34a-5p/Sirt1/p66shc pathway contributes to doxorubicin-induced cardiotoxicity. Sci. Rep. 7, 11879. 10.1038/s41598-017-12192-y 28928469 PMC5605522

