# Supplementary figures and images for: Cardiac miRNA expression during the development of chronic anthracycline-induced cardiomyopathy using an experimental rabbit model
Source: Front Pharmacol. 2024 Jan 3;14:1298172. doi: 10.3389/fphar.2023.1298172 (PMC10791979; doi:10.3389/fphar.2023.1298172)

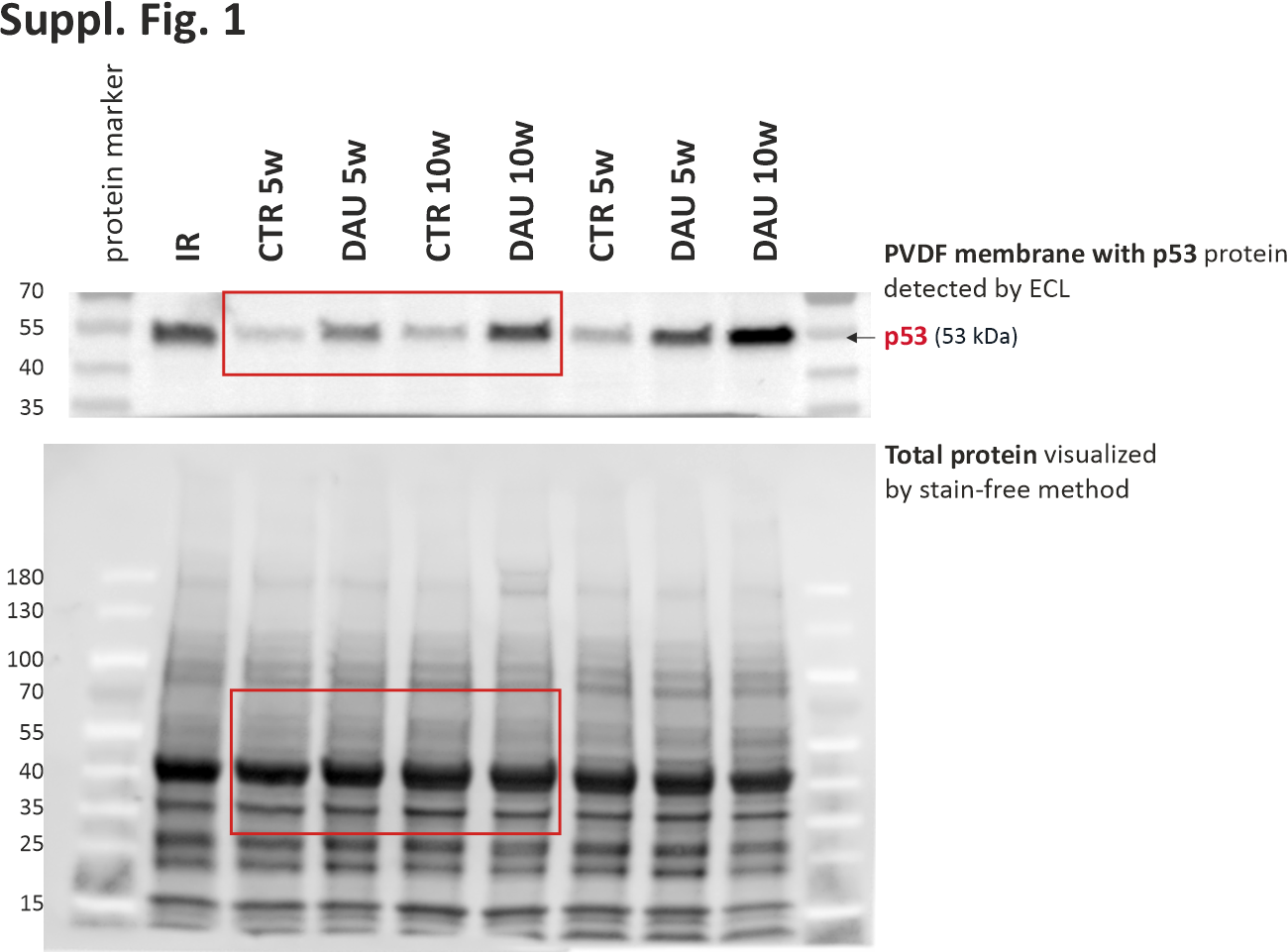

Supplement: Supplementary file 1 [file Image1.TIF]
